# Natural active herbal monomers for the treatment of thromboembolic diseases: a review

**DOI:** 10.3389/fphar.2025.1607415

**Published:** 2025-09-03

**Authors:** Zhou-Yu Nie, Jia-Qi Zhang, Yuan-Jia-Yi Shen, Jia-Qi Xi, Yong-Bing Cao, Li-Chao Zhang, Ling Li

**Affiliations:** ^1^ Institute of Vascular Disease, Shanghai TCM-Integrated Hospital, Shanghai University of Traditional Chinese Medicine, Shanghai, China; ^2^ Department of Pharmacy, Shanghai Municipal Hospital of Traditional Chinese Medicine, Shanghai, China

**Keywords:** thromboembolism, thrombosis, antithrombosis, natural herbal monomers, small molecule compounds

## Abstract

Thromboembolism is a leading cause of morbidity and mortality worldwide. Current methods of treating thromboembolism include anticoagulant therapy, thrombolytic therapy, and surgical removal of the thrombus. All of these treatments have some drawbacks, such as an increased risk of bleeding, limitation to fresh thrombus, and a high recurrence rate. Therefore, there is an urgent need to find effective and safe drugs for the treatment of thromboembolism. In recent years, it has been found that many natural active herbal monomers exhibit distinct advantages in treating this condition. In this review, the therapeutic effects of effective active monomers from natural herbs on thromboembolism, including flavonoids, polyphenols, alkaloids, terpenoids, saponins, and organic acids, were described. Furthermore, their antioxidant, anti-inflammatory, inhibition of platelet aggregation and antithrombotic effects through nuclear factor NF-κB, ERK1/2, PI3K, Akt and other signaling pathways were systematically summarized. Altogether, this review provides a comprehensive summary of promising therapeutic candidate drugs for the treatment of thromboembolic diseases and aims to guide future preclinical and clinical research for novel, safe and effective antithrombotic therapies.

## 1 Introduction

Thromboembolic diseases refer to a series of diseases caused by vascular obstruction due to thrombosis or thrombus embolization, such as ischemic stroke, acute myocardial infarction, and deep vein thrombosis. The American Society of Hematology reports that VTE occurs in 1–2 individuals per 1,000 each year, or ∼300,000 to 600,000 events in the United States annually (Ortel et al., 2020), and acute venous and arterial thromboses account for the most common causes of death in developed countries (Ashorobi et al., 2025).

Current treatments for thromboembolism include anticoagulant therapy, thrombolytic therapy, mechanical thrombectomy, inferior vena cava filter placement, and other methods for treating thrombus (Sagris et al., 2022). However, all of these treatments have some drawbacks. Conventional antithrombotic therapy is primarily based on three classes of drugs: anticoagulants, antiplatelet agents, and thrombolytics. Anticoagulants, such as the vitamin K antagonist warfarin, heparins, and direct oral anticoagulants (DOACs) that inhibit Factor Xa or thrombin, are effective in preventing further expansion and propagation of the thrombus but carry a significant bleeding risk, require monitoring, and show a limited effect on the dissolution of thrombi that have already formed (Helin et al., 2019; Gailani and Gruber, 2024). Thrombolytic drugs such as recombinant tissue-type plasminogen activator (rt-PA) can rapidly dissolve thrombi but are limited to the acute phase of thrombosis (fresh thrombus formed within 3–4.5 h). Their short therapeutic window, high risk of bleeding complications, and limited patient suitability further restrict their use (Montalvan Ayala et al., 2022; Khedr et al., 2023). Surgical mechanical debridement and inferior vena cava filter placement can remove thrombus directly through catheters, but they are invasive, costly, and only suitable for large vessel obstructions due to the risks of infection or vascular damage (Goldhaber et al., 2021). Notably, a major limitation of these synthetic drugs is that they typically target a single molecule or pathway, which, while potent, contributes to their narrow therapeutic index and risk of side effects. Therefore, the search for new drugs for the prevention and treatment of thromboembolic diseases is urgent and necessary (Kim et al., 2021). Many natural herbal monomers, as reviewed herein, exhibit a multi-target approach (Mu et al., 2023; Yin et al., 2023). These natural compounds can simultaneously exert anti-inflammatory, antioxidant, antiplatelet, and anticoagulant effects, potentially offering a broader therapeutic window and a more favorable safety profile by modulating the entire thrombotic milieu rather than a single step. This review, therefore, aims to systematically evaluate these natural compounds to highlight their mechanisms in comparison to conventional therapies and identify promising candidates for future drug development.

An increasing number of studies have found that natural medicinal plants and their active monomers are widely used in a variety of diseases and have strong therapeutic potential. The efficacy of natural active herbal monomers in the treatment of thromboembolism is remarkable, effectively avoiding toxic side effects, reducing recurrence rates and improving the prognosis. Natural products have always been the cornerstone of modern drug discovery, providing lead compounds for many first-line clinical drugs (Newman and Cragg, 2020). Faced with the limitations of existing antithrombotic drugs, the search for new active monomers from medicinal herbs represents a promising frontier. However, despite numerous preclinical studies, translating these findings into clinical applications remains a challenge. Therefore, a systematic review of preclinical evidence is crucial for identifying the most promising drug candidates, elucidating their mechanisms of action, and providing a solid foundation for designing rigorous clinical trials in the future. Hence, the therapeutic effects of effective active monomers of natural herbal on thromboembolism, the molecular mechanisms and the research progress of related targets are reviewed. Therefore, this review aims to provide a scientific basis for the rational development of natural herbal monomers as complementary or alternative therapies, and to promote their translation from basic research to future clinical applications.

## 2 Natural herbal agents with effects on thromboembolism

**FIGURE 1:**
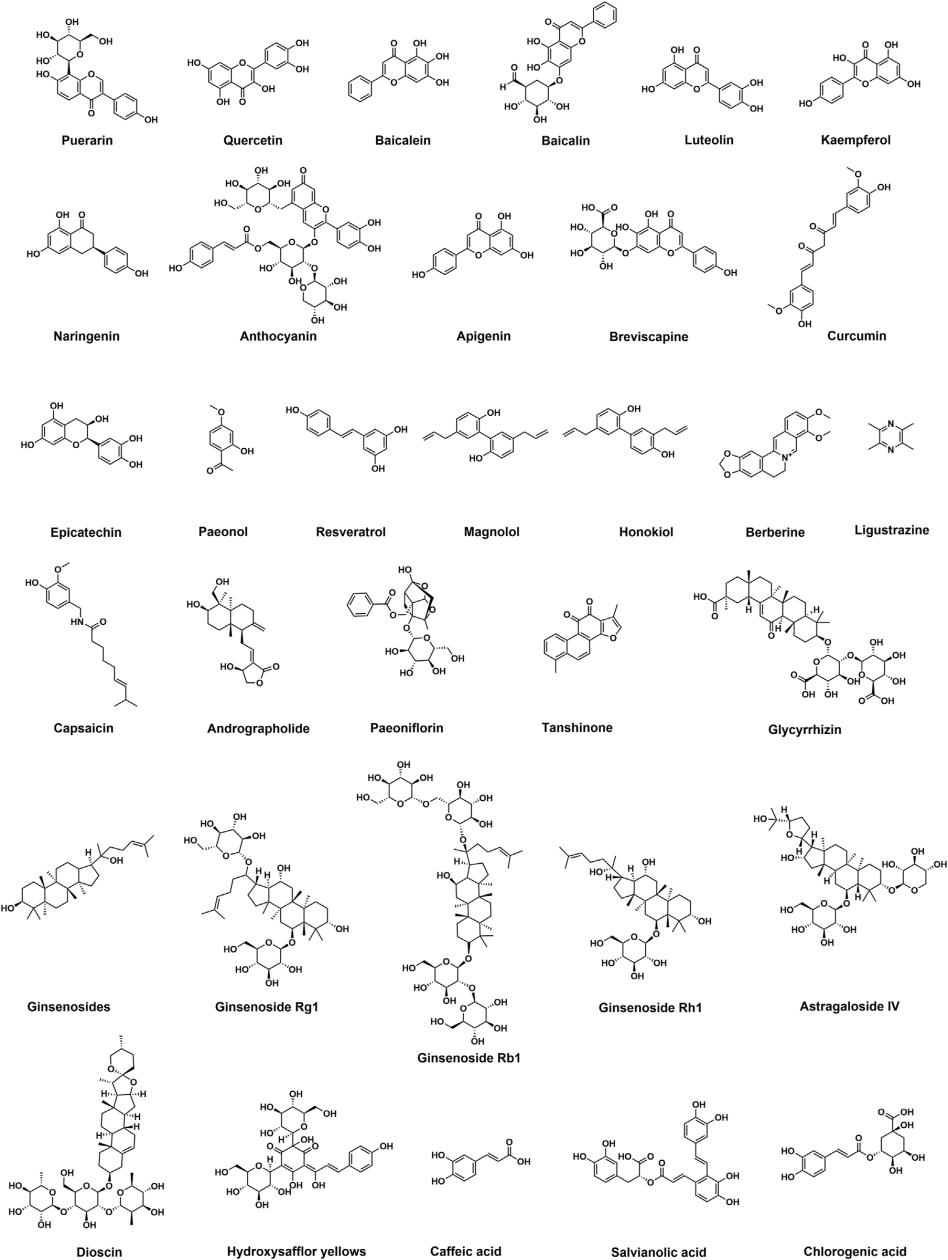
Chemical structures of representative antithrombotic natural active herbal monomers.

Natural active herbal monomers mainly include flavonoids, polyphenols, terpenoids, alkaloids, saponins and organic acids (The chemical structure and molecular formula of representative compounds were shown in Figure 1; Table 1) which have anti-inflammatory, antioxidant, antiplatelet and antithrombotic effects and are considered as potential drugs for the treatment of thromboembolic diseases (Mu et al., 2023; Yin et al., 2023).

**TABLE 1:** Flavonoids, polyphenols, alkaloids, terpenoids, saponin and organic acids.

	Compound	Molecular formula	Molecular mass	PubChem Cid
Puerarin	7,4′-dihydroxy-8-c-glycosylisoflavone	C_21_H_20_O_9_	416.37	5281807
Quercetin	3,3,4,5,7-pentahydroxyflavone	C_15_H_10_O_7_	302.23	5280343
Baicalein	5,6,7-trihydroxy-2-phenyl-4H-chromen-4-one	C_15_H_10_O_5_	270.24	5281605
Baicalin	7-D-Glucuronic acid-5,6-dihydroxyflavone	C_21_H_18_O_11_	446.4	64982
Luteolin	3′,4′,5,7-Tetrahydroxyflavone	C_15_H_10_O_6_	286.24	5280445
Kaempferol	3,5,7-trihydroxy-2-(4-hydroxyphenyl)chromen-4-one	C_15_H_10_O_6_	286.24	5280863
Naringenin	(2S)-5,7-dihydroxy-2-(4-hydroxyphenyl)-2,3-dihydrochromen-4-one	C_15_H_12_O_5_	272.25	439246
Anthocyanin	Cyanidin 3-O-[2″-O-(xylosyl)-6″-O-(p-coumaroyl) glucoside] 5-O-glucoside cyanidin 3-O-[6-O-(p-coumaroyl)-2-O-(beta-D-xylosyl)-beta-D-glucosyl]-5-O-beta-D-glucoside	C_41_H_44_O_22_	888.8	145865157
Apigenin	5,7-Dihydroxy-2-(4-hydroxyphenyl)-4H-chromen-4-one	C_15_H_10_O_5_	270.24	5280443
Breviscapine	(2S,3S,4S,5R,6S)-6-[5,6-dihydroxy-2-(4-hydroxyphenyl)-4-oxochromen-7-yl]oxy-3,4,5-trihydroxyoxane-2-carboxylic acid	C_21_H_18_O_12_	462.4	185617
Curcumin	(1E,6E)-1,7-bis(4-hydroxy-3-methoxyphenyl)hepta-1,6-diene-3,5-dione	C_21_H_20_O_6_	368.4	969516
Epicatechin	(2R,3R)-2-(3,4-dihydroxyphenyl)-3,4-dihydro-2H-chromene-3,5,7-triol	C_15_H_14_O_6_	290.27	72276
Paeonol	1-(2-hydroxy-4-methoxyphenyl)ethanone	C_9_H_10_O_3_	166.17	11092
Resveratrol	5-[(E)-2-(4-hydroxyphenyl)ethenyl]benzene-1,3-diol	C_14_H_12_O_3_	228.24	445154
Magnolol	2-(2-hydroxy-5-prop-2-enylphenyl)-4-prop-2-enylphenol	C_18_H_18_O_2_	266.3	72300
Honokiol	2-(4-hydroxy-3-prop-2-enylphenyl)-4-prop-2-enylpheno	C_18_H_18_O_2_	266.3	72303
Berberine	16,17-dimethoxy-5,7-dioxa-13-azoniapentacyclo [11.8.0.02,10.04,8.015,20]henicosa-1(13),2,4(8),9,14,16,18,20-octaene	C_20_H_18_NO_4_^+^	336.4	2353
Capsaicin	(E)-N-[(4-hydroxy-3-methoxyphenyl)methyl]-8-methylnon-6-enamide	C_18_H_27_NO_3_	305.4	1548943
Ligustrazine	2,3,5,6-tetramethylpyrazine	C_8_H_12_N_2_	136.19	14296
Andrographolide	(3E,4S)-3-[2-[(1R,4aS,5R,6R,8aS)-6-hydroxy-5-(hydroxymethyl)-5,8a-dimethyl-2-methylidene-3,4,4a,6,7,8-hexahydro-1H-naphthalen-1-yl]ethylidene]-4-hydroxyoxolan-2-one	C_20_H_30_O_5_	350.4	5318517
Paeoniflorin	[(1R,2S,3R,5R,6R,8S)-6-hydroxy-8-methyl-3-[(2S,3R,4S,5S,6R)-3,4,5-trihydroxy-6-(hydroxymethyl)oxan-2-yl]oxy-9,10-dioxatetracyclo [4.3.1.02,5.03,8]decan-2-yl]methyl benzoate	C_23_H_28_O_11_	480.5	442534
Tanshinone	1,6-dimethylnaphtho [1,2-g][1]benzofuran-10,11-dione	C_18_H_12_O_3_	276.3	114917
Glycyrrhizin	(2S,3S,4S,5R,6R)-6-[(2S,3R,4S,5S,6S)-2-[[(3S,4aR,6aR,6bS,8aS,11S,12aR,14aR,14bS)-11-carboxy-4,4,6a,6b,8a,11,14b-heptamethyl-14-oxo-2,3,4a,5,6,7,8,9,10,12,12a,14a-dodecahydro-1H-picen-3-yl]oxy]-6-carboxy-4,5-dihydroxyoxan-3-yl]oxy-3,4,5-trihydroxyoxane-2-carboxylic acid	C_42_H_62_O_16_	822.9	14982
Ginsenosides	(3S,5R,8R,9R,10R,14R,17S)-17-(2-hydroxy-6-methylhept-5-en-2-yl)-4,4,8,10,14-pentamethyl-2,3,5,6,7,9,11,12,13,15,16,17-dodecahydro-1H-cyclopenta [a]phenanthren-3-ol	C_30_H_52_O_2_	444.7	3086007
Astragaloside IV	(2R,3R,4S,5S,6R)-2-[[(1S,3R,6S,8R,9S,11S,12S,14S,15R,16R)-14-hydroxy-15-[(2R,5S)-5-(2-hydroxypropan-2-yl)-2-methyloxolan-2-yl]-7,7,12,16-tetramethyl-6-[(2S,3R,4S,5R)-3,4,5-trihydroxyoxan-2-yl]oxy-9-pentacyclo [9.7.0.01,3.03,8.012,16]octadecanyl]oxy]-6-(hydroxymethyl)oxane-3,4,5-trio	C_41_H_68_O_14_	785.0	13943297
Dioscin	(2S,3R,4R,5R,6S)-2-[(2R,3S,4S,5R,6R)-4-hydroxy-2-(hydroxymethyl)-6-[(1S,2S,4S,5′R,6R,7S,8R,9S,12S,13R,16S)-5′,7,9,13-tetramethylspiro [5-oxapentacyclo [10.8.0.02,9.04,8.013,18]icos-18-ene-6,2′-oxane]-16-yl]oxy-5-[(2S,3R,4R,5R,6S)-3,4,5-trihydroxy-6-methyloxan-2-yl]oxyoxan-3-yl]oxy-6-methyloxane-3,4,5-triol	C_45_H_72_O_16_	869.0	119245
Hydroxysafflor yellows	(6E)-2,5-dihydroxy-6-[(E)-1-hydroxy-3-(4-hydroxyphenyl)prop-2-enylidene]-2,4-bis [(2S,3R,4R,5S,6R)-3,4,5-trihydroxy-6-(hydroxymethyl)oxan-2-yl]cyclohex-4-ene-1,3-dione	C_27_H_32_O_16_	612.5	6443665
Caffeic acid	(E)-3-(3,4-dihydroxyphenyl)prop-2-enoic acid	C_9_H_8_O_4_	180.16	689043
Salvianolic acid	(2R)-3-(3,4-dihydroxyphenyl)-2-[(E)-3-[2-[(E)-2-(3,4-dihydroxyphenyl)ethenyl]-3,4-dihydroxyphenyl]prop-2-enoyl]oxypropanoic acid	C_26_H_22_O_10_	494.4	
Chlorogenic acid	(1S,3R,4R,5R)-3-[(E)-3-(3,4-dihydroxyphenyl)prop-2-enoyl]oxy-1,4,5-trihydroxycyclohexane-1-carboxylic acid	C_16_H_18_O_9_	354.31	1794427

### 2.1 Flavonoids

Flavonoids have a basic structural unit of 2-phenylchromone, including puerarin, quercetin, baicalein, baicalin, luteolin, kaempferol, naringenin, anthocyanins, apigenin and breviscapine, with anti-inflammatory, antioxidant, antibacterial effects, cardiovascular protection and thrombosis prevention (Chen et al., 2023).

#### 2.1.1 Puerarin

Puerarin is an isoflavone isolated from the roots of *Pueraria lobata*, which has a variety of pharmacological activities such as anti-inflammatory, antioxidant, inhibition of apoptosis (Jiang et al., 2022). A previous study found that *in vitro* puerarin and its metabolite daidzein inhibited adenosine diphosphate (ADP) and collagen-induced platelet aggregation, thereby exerting antithrombotic effects (as shown in Table 2) (Choo et al., 2002). A recent study found that *in vitro* pretreatment of human umbilical vein endothelial cells (HUVECs) with puerarin for 1 h significantly attenuated oxidized low-density lipoprotein (ox-LDL) induced tissue factor (TF) expression, enhanced protein kinase B (Akt) phosphorylation and nitric oxide (NO) production, and inhibited extracellular signal-regulated kinase 1/2 (ERK1/2) and nuclear factor Kappa B (NF-κB) activation, suggesting that puerarin has anticoagulant effects and is a potential drug for coronary artery disease and thrombosis prevention (Deng et al., 2017). It has also been shown that *Gegen Qinlian* pills (the main ingredient is Pueraria lobata, each gram of *Gegen Qinlian* pills contains 2.78 mg of pueraria lobata) through modulation of the HMGB1/NF-κB/NLRP3 signaling pathway decreased tumor necrosis factor-α (TNF-α) in plasma and high mobility group protein 1 (HMGB1) in lung tissue, and thereby inhibiting carrageenan induceed pulmonary, hepatic, and caudal thrombosis and increased caudal blood flow (Wei et al., 2022).

**TABLE 2:** Flavonoids are used in the treatment of thromboembolism.

Chemical structure	Drug	Animal model	Dose and administration	Effect and mechanism	References
Flavonoids	Puerarin	Collagen (110 mg) and epinephrine (13 mg) induced pulmonary thrombosis in mice	25, 50 mg/kg; IG	↓ platelet aggregation	Choo et al. (2002)
	Quercetin	Thrombin (3300 NIH U/mg) induced acute thromboembolism model in mice	1, 5, 10 and 20 mg/kg; IG	↓ Thrombin and FXa activity; Fibrin clot; Blood clotting; Platelet activation and aggregation	Choi et al. (2016)
		FeCl_3_ (20%) induced carotid injury model in mice	6 mg/kg; IP for 7 days or IV for once	↓ Platelet aggregation; Platelet granule exocytosis; Vessel occlusion ↑ Artery blood flow	Mosawy et al. (2013)
		FeCl_3_ (10%) induced carotid thrombosis model in mice	50 and 100 mg/kg; IG; Twice a day for 3 days	↓ ATP; α_IIb_β_3_; P-selectin; Ca^2+^; ROS; Platelet aggregation; GPVI; pSyk, pPLCγ2, pPI3K, pAKT, pTRAF4, p47^phox^, pHic5; Infarct volume in stroke ↑ PTP dephosphorylation; Tail bleeding times	Oh et al. (2021)
		Ischemia and reperfusion induced stroke in mice	50 and 100 mg/kg; IG; Twice a day for 3 days		
		Micropoint ablation laser injured testicle arteriole walls induced arterial thrombosis model in mice	200 mg/kg; IG; Twice a day for 2 days	↓ Platelet aggregation; Granule secretion; Integrin α_IIb_β_3_ function; Ca^2+^; pSyk and LAT	Stainer et al. (2019)
	Baicalin	FeCl_3_ (0.25 mol/L) induced testicular artery thrombosis model in mice	0.89 and 2.23 mg/kg; IV	↓ Thrombin-catalyzed fibrin polymerization; Thrombin and FXa; PAI-1 ↑ APTT and PT; Bleeding time	Lee et al. (2015)
		Stenosis of IVC induced DVT model in rats	80 mg/kg; IG	↓ SIRT; Thrombus weight ↑ NF-κB; Migratory and angiogenetic abilities of EPCs	Xie et al. (2025)
	Luteolin	FeCl_3_ (7.5%) induced mesenteric thrombosis in mice	35 μM/kg; IP	↓ Platelet aggregation, adhesion; ROS; ITAM, MAPK, GPVI; Integrin α_IIb_β_3_, oxidative stress ↑ Platelet endogenous antioxidant capacity Not affect coagulation, hemostasis, or platelet production	Ye et al. (2023)
		Collagen and epinephrine induced acute pulmonary embolism model in mice	35 μM/kg; IP		
		FeCl_3_ (4%) induced carotid arterial thrombosis in mice	10 and 20 mg/kg	↓ Thrombin and FXa activity; Fibrin polymer formation ↑ APTT and PT	Choi et al. (2015a)
	Kaempferol	Collagen (250 μg/mL) and epinephrine (150 μg/mL) and thrombin (3300 NIH U/mg) induced acute thromboembolism models in mice	5, 10 and 20 mg/kg; IP	↓ Thrombin and FXa; Fibrin polymer formation; pERK1/2, p38, pJNK1/2, pPI3K and pAKT	Choi et al. (2015b)
		FeCl_3_ (4%) induced carotid arterial thrombus model in rats	5, 10 and 20 mg/kg; IP		
		Electrocoagulation (1.00 mA for 5 min) induced autologous thrombus stroke model in rats	50 mg/kg/d; IP; For 14 days	↓ Neurological deficits; Infarct volume; Vascular embolization ↑ MECs survival, proliferation, migration, lumen formation; Neovascular; TJPs, HIF-1α/VEGF-A/Notch1	Zhang et al. (2025)
		FeCl_3_ (20%) induced carotid arterial thrombosis in mice	25 and 50 mg/kg	↓ Platelet aggregation and adhesion; Superoxide anion generation; p47, pSyk, pBtk, pPLCγ2, pVav1; pNOX; Ca^2+^; P-selectin; α_IIb_β_3_ ↑ SHP-2	Wang et al. (2015)
	Naringenin	FeCl_3_ (10%) induced carotid thrombosis model in rats	200, 400 and 800 mg/kg	↓ Platelet aggregation, adhesion; α-granule; Fibrinogen binding; Ca^2+^; Fibrinogen, clot retraction; PI3K; Phosphodiesterase ↑ cGMP; VASP^Ser239^	Huang et al. (2021)

Note: ↑, increase or enhance; ↓, decrease or inhibit.

Abbreviations: IVC, inferior vena cava; DVT, deep vein thrombosis; IG, intragastric administration; IV, intravenous injection; IP, intraperitoneal injection; FXa, Factor Xa; α_IIb_β_3_, alpha IIb, beta 3 integrin; ROS, reactive oxygen species; GPVI, Glycoprotein VI; syk, Spleen tyrosine kinase; PLCγ2, Phospholipase Cγ2; PI3K, Phosphoinositide 3-kinase; AKT, Protein kinase B; TRAF4, TNF, receptor-associated factor 4; p47^phox^, Phagocyte oxidase 47-kilodalton protein; PTPs, Protein tyrosine phosphatase; LAT, Linker for activation of T cells; PAI-1, Plasminogen activator inhibitor-1; SIRT, sirtuin; ITAM, immunoreceptor tyrosine based activation motif; MAPK, mitogen activated protein kinase; ERK1/2, Extracellular signal regulated kinase 1/2; p38, p38 mitogen activated protein kinase; JNK1/2, c-Jun N-terminal kinase 1/2; MECs, TJPs, Tight-junction proteins; HIF-1α, Hypoxia inducible factor 1 Alpha; VEGF-A, Vascular endothelial growth factor A; Notch1, Neurogenic locus notch homolog protein 1; Btk, Bruton’s tyrosine kinase; Vav1, Vav guanine nucleotide exchange factor 1; NOX, NADPH, oxidase; SHP-2, Src homology 2 domain-containing protein tyrosine phosphatase-2; cGMP, cyclic guanosine monophosphate; VASP^Ser239^, Vasodilator stimulated phosphoprotein, serine 239; NF-κB, Nuclear factor kappa-light-chain-enhancer of activated B cells; APTT, activated partial thromboplastin time; PT, prothrombin time; Microvascular endothelial cells.

#### 2.1.2 Quercetin

Quercetin, a flavonoid widely distributed in nature and the human diet and particularly abundant in onions, exhibits potent anti-inflammatory and antioxidant bioactivities (Singh et al., 2021; Qi et al., 2022). It has been shown that quercetin inhibited thrombin and coagulation factor Xa (FXa) activity in a thrombin-induced acute thromboembolism model in mice, thereby inhibiting thrombus formation (Choi et al., 2016). It has also been shown that quercetin inhibited ferric chloride (FeCl_3_)-induced carotid artery thrombosis in mice, and inhibited platelet aggregation and platelet dense granule secretion, improved carotid blood flow, and thus exerted antiplatelet and thrombopreventive effects (Mosawy et al., 2013; Oh et al., 2021). It was found that quercetin and two of its methylated metabolites, isorhamnetin and tamarixetin, inhibited arterial thrombosis caused by laser injury in mice and interacted with aspirin to enhance antiplatelet effects (as shown in Table 2) (Stainer et al., 2019). A clinical trial reported that isoquercetin at a dose of 1 g day^-1^ for 56 days significantly reduced D-dimer, P-selectin, and platelet-dependent fibrin production in cancer patients compared to placebo, suggesting that supplementation with isoquercetin prevents hypercoagulability and thrombosis in cancer patients (Manjunath and Thimmulappa, 2022). A multicenter, double-blind phase Ⅲ trial is now evaluating isoquercetin 1 g day^-1^ for primary VTE prophylaxis in metastatic pancreatic cancer (ClinicalTrials.gov Identifier: NCT06861088, accessed 9 July 2025). Consistent benefits were already reported in the CATIQ phase Ⅱ study, where isoquercetin 1,000 mg day^-1^ reduced D-dimer and platelet-dependent fibrin formation across 72 patients with advanced solid tumours (ClinicalTrials.gov Identifier: NCT02195232).

#### 2.1.3 Baicalein and baicalin

Baicalein and baicalin are typical flavonoids extracted from the plant *Scutellaria baicalensis*, which have a variety of medicinal properties such as anti-inflammatory, antiviral, antibacterial and hypoglycemic effects (Zhao et al., 2022). It was shown that a deep vein thrombosis model was prepared by incompletely ligating the inferior vena cava (IVC) of rats, and baicalein was found to inhibit thrombosis by promoting the migration of endothelial progenitor cells (EPCs) and angiogenesis through SIRT1/NF-κB signaling (Xie et al., 2025). It was found that baicalin inhibited FeCl_3_-induced arterial thrombosis, significantly prolonged the activated partial thromboplastin time (APTT) and plasminogen time (PT), decreased the ratio of plasminogen activator inhibitor type 1 to tissue-type plasminogen activator (PAI-1/t-PA), and inhibited the activities of thrombin and coagulation factor FXa, inhibited thrombin-catalyzed fibrin polymerization and platelet function, thereby exerting antithrombotic effects (as shown in Table 2) (Lee et al., 2015).

#### 2.1.4 Luteolin

Luteolin is a flavonoid polyphenolic compound that are widely found in fruits, vegetables, flowers and herbs such as honeysuckle and Perilla, and exhibit a variety of pharmacological activities including anti-inflammatory, antioxidant and antitumor activities (Zhu et al., 2024). It was shown that luteolin exhibited potent antithrombotic activity by inhibiting IgG-like receptor glycoprotein VI (GPVI) and thereby inhibiting FeCl_3_-induced mesenteric artery thrombosis, inhibiting collagen and convulxin-induced platelet aggregation and adhesion, and decreasing oxidative stress (Ye et al., 2023). It has also been shown that luteolin inhibited FeCl_3_-induced carotid artery thrombosis, suppressed oxidative stress, inhibited thrombin activity, prolonged APTT and PT, and reduced thrombosis (as shown in Table 2) (Choi et al., 2015a).

#### 2.1.5 Kaempferol

Kaempferol is a flavonoid found in the tea plant (Camelia sinensis) with pharmacological activities such as hepatoprotective, antibacterial and antidiabetic properties (Periferakis et al., 2022). It was shown that kaempferol inhibited thrombus formation in three animal models of thrombosis (collagen-adrenaline and thrombin-induced acute thromboembolism model and FeCl_3_-induced carotid artery thrombosis model) and significantly suppressed the activity of prothrombin and coagulation factor FXa and inhibited the formation of fibrin polymers (Choi et al., 2015b). It has also been shown that kaempferol reduced cerebral infarct size and promoted neovascularization and vascular remodeling in a rat cerebral thrombotic stroke model through the HIF-1α/VEGF-A/Notch1 pathway, suggesting that kaempferol has therapeutic potential for the treatment of ischemic stroke (Zhang et al., 2025). In addition, it was found that kaempferol inhibited collagen-induced platelet activation, aggregation and adhesion by inhibiting nicotinamide adenine dinucleotide phosphate (NADPH) oxidase and protecting src homology 2 domain-containing protein tyrosine phosphatase-2 (SHP-2) from oxidative inactivation *in vitro*, suggesting that kaempferol has therapeutic potential for the prevention and treatment of thrombosis and cardiovascular diseases (as shown in Table 2) (Wang et al., 2015).

#### 2.1.6 Naringenin

Naringenin belongs to the polyphenol flavanone family, mainly found in citrus fruits such as grapefruit and medicinal plants, with bioactivities such as anti-inflammatory, antioxidant, neuroprotective, hepatoprotective and anti-cancer properties (Motallebi et al., 2022). It was reported that naringin was found to inhibit FeCl_3_-induced carotid artery thrombosis in rats by inhibiting phosphatidylinositol 3-kinase (PI3K) and cyclic nucleotide signaling, without affecting bleeding time. In addition, it was found that naringenin dose-dependently inhibited ADP induced platelet aggregation and adhesion, and inhibited platelet α-granule secretion, fibrinogen binding, and intracellular calcium mobilization *in vitro*, indicating that naringin has antithrombotic effects (as shown in Table 2) (Huang et al., 2021).

#### 2.1.7 Anthocyanin

Anthocyanins are a class of water-soluble flavonoids, widely found in food and plants, such as red cabbage microgreen, blueberry, blackcurrant, mulberry, cherry, black elderberry, black soybean, chokeberry and jaboticaba peel, which have been shown to improve cardiovascular disease and cognitive ability, and prevent neurodegenerative diseases (Mattioli et al., 2020; Avula et al., 2023). A clinical study showed that 320 mg of anthocyanins daily for 28 consecutive days significantly inhibited ADP-induced platelet aggregation and reduced the risk of thrombosis by lowering mean platelet volume (MPV), mean cellular hemoglobin (MCH) and fibrinogen levels in healthy subjects compared with placebo controls (ClinicalTrials.gov Identifier: ACTRN12615000293561) (Gaiz et al., 2022). In another clinical study, a patient with metabolic syndrome (MetS) took 320 mg of anthocyanins twice daily for 28 consecutive days. It turns out that in comparison with the placebo control group, his fasting blood glucose, triglyceride, low-density lipoprotein (LDL-C) and C-reactive protein (CRP) levels were reduced, and the ADP-induced platelet activation and p-selectin levels were inhibited, thereby decreasing cardiovascular risk factors and reducing thrombogenicity in a MetS population (ClinicalTrials.gov Identifier: ACTRN12615000293561) (Aboonabi et al., 2020).

#### 2.1.8 Apigenin

Apigenin is a natural flavonoid, and present principally as glycosylated in significant amount in vegetables (parsley, celery, onions) fruits (oranges), herbs (chamomile, thyme, oregano, basil), and plant-based beverages (tea, beer, and wine), with a variety of biological activities including anti-inflammatory, antiviral and anticancer properties (Liu et al., 2024). It was reported that apigenin inhibited thrombosis by repressing arachidonic acid (AA) metabolism, thereby inhibiting collagen-induced platelet aggregation and adhesion. Furthermore, apigenin was found to enhance the effect of aspirin on platelet aggregation (Navarro-Nunez et al., 2008).

#### 2.1.9 Breviscapine

Breviscapine is a flavonoid compound extracted from *Erigeron breviscapus*, a plant of the Asteraceae family, which has the effects of increasing blood flow, improving microcirculation, dilating blood vessels, reducing blood viscosity, promoting fibrinolysis, inhibiting platelet aggregation and thrombosis, *etc.* (Wen et al., 2021). It was shown that breviscapine exerted its anticoagulant effect by reducing clotting time (CT) and prothrombin time (PT), inhibiting platelet factor III (PF3) activity, and decreasing euglobulin cleavage time (ELT) (Wang et al., 2003).

### 2.2 Polyphenols

The central feature of polyphenols is the presence of one or more benzene rings (aromatic rings) and hydroxyl groups attached to the benzene rings. Polyphenols include curcumin, epicatechin, paeonol, resveratrol, magnolol and honokiol, which have antioxidant, anti-inflammatory, and anticancer properties.

#### 2.2.1 Curcumin

Curcumin, derived from the rhizome of the spice turmeric (*Curcuma longa L.*), is a naturally occurring polyphenolic compound with anti-inflammatory, antioxidant, thromboprophylactic, and cardiovascular protective properties (Keihanian et al., 2018; Abd El-Hack et al., 2021). A previous study reported that curcumin promoted venous thrombus resolution in mice by modulating the miR-499-mediated PTEN/VEGF/Ang-1 signaling pathway (Wang T. et al., 2021). Curcumin was found to reduce miR-21 expression by down-regulating specificity protein 1 (Sp1) and up-regulating phosphatase and tensin homolog (PTEN) and inhibiting the NF-κB signaling pathway, decreasing inflammatory factors and lung thrombus volume in an acute pulmonary embolism model in rats, thereby alleviating pulmonary thromboembolism (Liang et al., 2021). It has also been shown that curcumin reduced cerebral infarct volume and edema volume and increased glutathione peroxidase (GSH-Px) levels in a dose-dependent manner in a rat thromboembolic stroke model, thereby ameliorating cerebral embolism and protecting cerebral nerves (as shown in Table 3) (Dohare et al., 2008). Other studies also found that curcumin inhibited GPVI-mediated platelet activation by inhibiting spleen tyrosine kinase (Syk) and phospholipase Cγ2 (PLCγ2) enzyme activities *in vitro* (Mayanglambam et al., 2010).

**TABLE 3:** Polyphenols are used in the treatment of thromboembolism.

Chemical structure	Drug	Animal model	Dose and administration	Effect and mechanism	References
Polyphenols	Curcumin	FeCl_3_ (15%) induced IVC thrombosis model in mice	1,000 mg/kg; Once daily for 14 days	↓ MiR-499 ↑ Angiogenesis; VEGF; Ang-1; HUVECs proliferation and migration	Wang et al. (2021c)
		Autogeneic thrombus injected into the right common jugular vein induceed APE model in rats	100 mg/kg; IG; Once daily for 45 days	↓ Sp1; miR-21; NF-κB ↑ PTEN; mPAP; RVSP; Wet weight/dry weight ratio, thrombus volume in the lungs	Liang et al. (2021)
		Autologous fibrin-rich clots injected into the external carotid artery one after another induceed embolic cerebral ischemia model in rats	100, 200 and 300 mg/kg; IP	↓ Edema volume; Neurological deficits; Neutrophil infiltration; Nitrite; Peroxynitrite; ROS; NO; NO synthase expression ↑ GSH; GSH-Px	Dohare et al. (2008)
	Epigallocatechin-3-Gallate	Stenosis of IVC induced DVT model in mice	25 mg/kg; IV	↓ Apoptosis; Iron and ROS ↑ EPCs proliferation, migration, and angiogenesis; ALOX15, ACSL4, FTH1; Nrf2, Slc7A11, HO-1 GPX4	Li et al. (2025)
	Paeonol	Thrombosis model in rats	1.25 mg/kg; IG; Once every other day; For 2 weeks	↓ Fibrinogen; D-dimer; TXB_2_ ↑ 6-keto-PGF_1α_ fibronectin; VEGF_165_; ERK1/2	Ye et al. (2016)
		AA (80 μM) induced thrombosis in zebrafish	1, 5, 10 and 20 μg/mL	↓ F2, FGA, FGB, vWF, PTGS1, TBXAS1; The inflammatory reaction, coagulation cascade reaction, and AA metabolism pathways	Lin et al. (2024)
	Resveratrol	IVC ligation induced venous thrombosis models in immuno deficient male nude rats	EPCs pre-treated by resveratrol (50 μM) were injected into the rats by IV	↓ MiR-138 ↑ EPCs migration and tube formation; FAK	Zhang et al. (2018)
		IVC ligation induced venous thrombosis models in immuno deficient male nude rats	EPCs pre-treated by resveratrol (25 μM) were injected into the rats by IV	↓ MiR-542-3p ↑ Angiogenic function of EPCs; ANGPT2	Lu et al. (2019)
	Magnolol	Fluorescein sodium (15 μg/kg) induced mesenteric microvessel thrombosis in mice	15 mg/kg; IP	↓ Platelet aggregation; Ca^2+^; PKCα; TXB_2_; COX-1 ↑ PPAR-β/γ; NO; GMP/PKG; pAkt; eNOS activity	Shih and Chou (2012)
	Honokiol	Electric current (3 mA) for 3 min induced carotid thrombosis model in rats	0.5, 5, 50 μg/kg; IV	↓ Platelet aggregation ↑ 6-keto-PGF_1α_; NO; PGI_2_	Hu et al. (2005)
		Fluorescein sodium (15 μg/kg) induced mesenteric microvessel thrombosis in mice	0.5 and 1 mg/kg	↓ Platelet aggregation; Ca2^+^; pLyn, pPLCγ2, pPKC, pMAPKs, pAkt; GPVI ↑ Closure time and occlusion time	Lee et al. (2017)

Note: ↑, increase or enhance; ↓, decrease or inhibit.

Abbreviations: IVC, inferior vena cava; DVT, deep vein thrombosis; APE, acute pulmonary embolism; AA, arachidonic acid; IG, intragastric administration; IV, intravenous injection; IP, intraperitoneal injection; TXB_2_, Thromboxane B_2_; 6-keto-PGF1α, 6-keto-prostaglandin F1 alpha; FAK, focal adhesion kinase; ANGPT2, Angiopoietin-2; PKCα, Protein kinase C alpha; COX-1, Cyclooxygenase-1; PGI2, prostacyclin; Lyn, LYN, proto-oncogene; Src family tyrosine kinase; PLCγ2, Phospholipase Cγ2; MAPK, mitogen activated protein kinase; AKT, Protein kinase B; GPVI, Glycoprotein VI; PPAR-β/γ, Peroxisome proliferator activated receptor beta/gamma; NO, nitric oxide; GMP, guanosine monophosphate; PKG, Protein kinase G; eNOS, endothelial nitric oxide synthase; Ang-1, Angiopoietin-1; NF-κB, Nuclear factor kappa-light-chain-enhancer of activated B cells; VEGF, vascular endothelial growth factor; Sp1, Specificity protein 1; F2, Coagulation factor II; FGA, fibrinogen alpha chain; FGB, fibrinogen beta chain; vWF, von willebrand factor; PTGS1, Prostaglandin endoperoxide synthase 1; TBXAS1, Thromboxane A Synthase 1; PTEN, phosphatase and tensin homolog; GSH, glutathione; GSH-Px, Glutathione peroxidase; ALOX15, Arachidonate 15-lipoxygenase; ACSL4, Acyl-CoA, synthetase long chain family member 4; FTH1, Ferritin Heavy Chain 1; Nrf2, Nuclear factor erythroid 2-related factor 2; Slc7A11, Solute Carrier Family 7 Member 11; HO-1, Heme Oxygenase-1; GPX4, Glutathione Peroxidase 4; HUVECs, Human umbilical vein endothelial cells; RVSP, right ventricular systolic pressure; mPAP, Mean pulmonary arterial pressure.

#### 2.2.2 Epicatechin

Epicatechin is derived from the herb catechu (*Acacia catechu (L. f.) Willd.*) and is a flavanol compound (Si et al., 2021). A study found that epicatechin inhibited platelet aggregation induced by ADP, thrombin, epinephrine, and collagen, and reduced clot lysis time (CLT) *in vitro*, indicating that epicatechin may have anticoagulant and cardiovascular preventive effects (Sinegre et al., 2019). Other studies have found that epigallocatechin-3-gallate promotes deep vein thrombosis resolution by regulating EPCs iron death via the nuclear factor erythroid 2-related factor 2 (Nrf2) pathway (as shown in Table 3) (Li et al., 2025).

#### 2.2.3 Paeonol

Paeonol is a natural active compound extracted from the root bark of peony (*Paeonia suffruticosa*), which has anti-inflammatory, prevention of cardiovascular disease, neuroprotective effects as well as other bioactive properties (Zhang L. et al., 2019). It was shown that paeonol promoted thrombus resolution by up-regulating the levels of phosphorylated ERK1/2 and increasing the expression level of vascular endothelial growth factor 165 (VEGF165) (Ye et al., 2016). It has also been found that paeonol combined with, geranylgeranyl-7-O-β-D-glucopyranoside and 5-hydroxymethylfurfural inhibited the inflammatory response, the coagulation cascade, and thus thrombosis in the AA-induced thrombus model in zebrafish, and the antithrombotic activity was most pronounced when the ratio was 4:3:3, indicating powerful prophylactic effect against thrombus (as shown in Table 3) (Lin et al., 2024).

#### 2.2.4 Resveratrol

Resveratrol is a phenolic substance isolated from *Veratrum grandiflorum*, which has cardiovascular disease prevention, anti-inflammatory, antioxidant and anti-aging properties (Breuss et al., 2019; Zhang et al., 2021). It was found that resveratrol inhibited inflammatory responses and thrombosis through inhibition of the HIF-1α/NLRP3 pathway in the model of stagnant deep vein thrombosis prepared by complete IVC ligation in rats (Fei et al., 2022). Similarly, in another study, resveratrol was found to improve EPCs function and promote venous thrombus resolution by upregulating adhesion plaque kinase (FAK) and inhibiting miR-138 in mice (Zhang et al., 2018). It was also found that resveratrol promoted neovascularization through inhibition of miR-542-3p and upregulation of angiopoietin-2 (ANGPT2) in rats, thereby promoting venous thrombus resolution (as shown in Table 3) (Lu et al., 2019). In brief, resveratrol not only prevented thrombosis but also promoted venous thrombus resolution.

#### 2.2.5 Magnolol and honokiol

Magnolol and honokiol are natural phenolic lignans isolated from *Magnolia officinalis*, which has anti-inflammatory, antioxidant, anticancer, neuroprotective, and cardiovascular protective effects (Zhang J. et al., 2019). It was reported that magnolol exerted cardiovascular modulatory effects especially strong therapeutic potential against atherosclerosis, thrombosis, hypertension and cardiac hypertrophy (Yuan et al., 2020). It was shown that magnolol inhibited platelet aggregation induced by collagen, AA, and thrombin, and suppressed the expression of thromboxane B_2_ (TXB_2_) and intracellular calcium mobilization, thereby exerting an antiplatelet effect (Teng et al., 1988). It was found that honokiol decreased electric current induced carotid thrombosis model in rats by upregulation of 6-keto-prostaglandin F1 alpha (6-keto-PGF_1α_). In a mouse model of mesenteric microvessel thrombosis induced by fluorescein sodium, honokiol downregulated Ca^2+^ mobilization and phosphorylation of LYN proto-oncogene (Lyn), PLCγ2, protein kinase C alpha (PKCα), mitogen activated protein kinase (MAPKs) and Akt to inhibite thrombosis (as shown in Table 3) (Lee et al., 2017).

### 2.3 Alkaloids

The core feature of alkaloids is the nitrogenous heterocyclic structure. Alkaloids include berberine, capsaicin, and ligustrazine, with anti-inflammatory, antioxidant, antiviral, anticancer and other pharmacological activities.

#### 2.3.1 Berberine

Berberine is an isoquinoline alkaloid isolated from the Chinese herb *Coptis chinensis Franch* and other berberis plants, which is used in the treatment of many diseases such as cancer and digestive, metabolic, cardiovascular, and neurological diseases (Song et al., 2020). It was also found that berberine inhibited FeCl_3_-induced carotid thrombosis by inhibiting pyruvate kinase muscle isozyme 2 (PKM2) to activate the t-PA-induced fibrinolytic system (Sun et al., 2024). Similarly, berberine inhibited ADP-induced platelet activation and keratin-induced thrombosis in mice by inhibiting the PI3Kβ/Rasa3/Rap1 pathway (Wang C. et al., 2021). In addition, integrated metabolomics and molecular docking revealed that berberine, the major metabolite of berberine *in vivo*, inhibited keratine-induced thrombosis in the tail of mice by modulating the catalytic cycle of vitamin K (Wang et al., 2023). It was also shown that berberine inhibited thrombosis induced by intraperitoneal injection of carrageenan by promoting the degradation of phenylacetic acid through modulation of the intestinal flora (as shown in Table 4) (Zhang H. J. et al., 2024). Berberine inhibited FeCl_3_-induced carotid artery thrombosis by increasing *Genus Lactobacillus* levels, remodeling the intestinal microbiota, and inhibiting trimethylamine N-oxide generation (Xie et al., 2021). Furthermore, the APLABE-PCI study is prospectively assessing whether adjunctive berberine (0.3–0.6 g day^-1^) enhances platelet inhibition in patients receiving dual antiplatelet therapy post-PCI (ClinicalTrials.gov Identifier: NCT03378934).

**TABLE 4:** Alkaloids, terpenoids, saponin and organic acids are used in the treatment of thromboembolism.

Chemical structure	Drug	Animal model	Dose and administration	Effect and mechanism	References
Alkaloids	Berberine	FeCl_3_ (35%) induced carotid arterial thrombosis in rats	50 and 100 mg/kg; IV	↓ Thrombus area; PKM2 ↑ Thrombus clogging time; t-PA	Sun et al. (2024)
		1% carrageenan solution (20 mg/kg, IV) induced thrombosis in mice	50 and 100 mg/kg; IG	Altering intestinal microbiota composition and related metabolites to inhibit thrombosis ↓ Biosynthesis of phenylacetylglycine ↑ Phenylacetic acid degradation	Zhang et al. (2024a)
		FeCl_3_ (6.5%) induced carotid arterial thrombosis in rats	20 mg/kg; IP		
		FeCl_3_ (20%) induced abdominal arterial thrombosis in rats	150 mg/kg; IG		
		0.5% carrageenan solution (50 mg/kg, IP) induced thrombosis in mice	50, 100, and 200 mg/kg; IG	↓ α_IIb_β_3_; P-selectin; Fibrinogen bind to platelets; PI3K/Akt; Rasa3 membrane translocation and Rap1 activation; class I PI3Kβ	Wang et al. (2021a)
		0.5% carrageenan solution (50 mg/kg, IP) induced thrombosis in mice	50 and 100 mg/kg; IG	Regulate phenylalanine, tyrosine, tryptophan biosynthesis and ubiquinone and other terpenoid-quinone biosynthesis Regulating the vitamin K catalytic cycle	Wang et al. (2023)
		Adrenalin hydrochloride (45 μM) induced thrombosis in AB zebrafish	10, 20 and 40 μg/mL	↓ Endothelial injury; Antiapoptosis; MAPK; ROS; FGA, FGB, FGG, F7; vWF	Zhang et al. (2022b)
Terpenoids	Andrographolide	IVC ligation induced venous thrombosis models in mice	5 mg/kg; IP	↓ TF activity; p50; NF-κB	Li et al. (2009)
	Paeoniflorin	FeCl_3_ (50%) induced carotid arterial thrombosis in rats	5, 10 and 25 mg/kg; IV	↓ Platelet aggregation and activation; Ca^2+^; Dense and α-granule; GPIIb/IIIa activation and fibrinogen binding; vWF engaged to platelets ↑ Occlusion time	Ngo et al. (2019)
	Glycyrrhizin	Thrombosis on a cotton thread in an arteriovenous shunt in the rats	30, 90, 120, 180, 360 mg/kg; IV	↑ APTT	Mendes-Silva et al. (2003)
		IVC ligation induced venous thrombosis model in male rats	300 mg/kg	↓ Neutrophils ↑ P-and L-selectin mRNA	Nakata et al. (2008)
Saponin	Ginsenoside Rk1	Collagen (50 μg) and epinephrine (6 μg) induced acute pulmonary thromboembolism in mice	30 mg/kg; IP	↓ Ca^2+^; α_IIb_β_3_	Shin et al. (2021)
	Ginsenoside Rp3	Collagen (50 μg) and epinephrine (6 μg) induced acute pulmonary thromboembolism in mice	10 mg/kg; IP	↓ Platelet aggregation; Ca^2+^, ATP, P-selectin; fibrinogen binding to integrin α_IIb_β_3_, fibronectin adhesion, and clot retraction; pMAPK, pSrc, pPLCγ2; PI3K/Akt ↑ cAMP levels and pVASP	Irfan et al. (2018)
	Ginsenoside Rp1	Thrombosis on a cotton thread in an arteriovenous shunt in the rats	15, 30 and 50 mg/kg; IG	↓ Platelet aggregation; ATP; TXB, p-selectin; Ca^2+^; α_IIb_β_3_; p38^MAPK^; ERK2; Fyn, Lyn, Syk, LAT, PI3K, PLCγ2 ↑ cAMP levels	Endale et al. (2012)
	Astragaloside IV	IVC stenosis induced DVT model in rats	10 mg/kg; IG; Once daily for 14 days	↓ The infiltration of leukocytes; Proinflammatory cytokines ↑ Migrative and angiogenic functions of EPCs; PI3K/AKT	Lyu et al. (2024)
	Hydroxysafflor yellows	PHZ (3 μM) induced zebrafish thrombosis	10、50 and 100 μM	↑ Blood circulation	Wang et al. (2021b)
Organic Acids	Caffeic acid	Photochemically induced cerebral artery thrombosis model	0.25, 1.25 and 5 mg/kg; IV	↓ Platelet aggregation; P-selectin; ATP; Ca^2+^; α_IIb_β_3_; p38, ERK and JNK ↑ cAMP	Lu et al. (2015)
		Topical application 20 μL ADP (20 mM) induced cerebral venous thrombosis model in mice	5 mg/kg; 6 mL/kg/h; IV		
	Salvianolic acid	Photochemical injury induced mesenteric artery thrombosis model in mice	10 mg/kg/h; IV	↓ Platelet aggregation and adhesion; P-selectin; Platelet binding of fibrinogen; pPI3K, pAkt	Huang et al. (2010)
		Photochemically induced cremaster arterioles thrombosis model in mice	80 mL/kg, 0.01 mM; 10 mg/kg; IV	↓ Platelet activation and aggregation; Blood coagulation; Fibrin network structures; thrombin	Neves et al. (2024)
		FeCl_3_ (10%) induced carotid arterial thrombosis in mice			
	Chlorogenic acid	Photochemical injury induced mesenteric artery thrombosis model in mice	200 mg/kg; IP	↓ Platelet aggregation, adhesion; sP-selectin, sCD40L, CCL5 and IL-1β ↑ cAMP; PKA; Adenosine A_2A_	Fuentes et al. (2014)
		Collagen (25 mg) and epinephrine (15 mg) induced APTE in mice	5 and 10 mg/kg; IP	↓ Fibrin clot; Procoagulant proteases; Thrombin; FXa; FXIIIa; Blood clot ↑ APTT, PT, TT	Choi and Kim (2017)

Note: ↑, increase or enhance; ↓, decrease or inhibit.

Abbreviations: IVC, inferior vena cava; DVT, deep vein thrombosis; APTE, acute pulmonary thromboembolism embolism; IG, intragastric administration; IV, intravenous injection; IP, intraperitoneal injection; PHZ, phenylhydrazine; FXa, Factor Xa; αIIbβ3, alpha IIb, beta 3 integrin; ROS, reactive oxygen species; PI3K, Phosphoinositide 3-kinase; AKT, Protein kinase B; MAPK, mitogen activated protein kinase; t-PA, tissue plasminogen activator; PKM2, Pyruvate kinase muscle Isozyme 2; vWF, von willebrand factor; NF-κB, Nuclear factor kappa-light-chain-enhancer of activated B cells; ERK2, Extracellular signal regulated kinase 2; PLCγ2, Phospholipase Cγ2; cAMP, cyclic adenosine monophosphate; VASP, vasodilator stimulated phosphoprotein; ATP, adenosine triphosphate; Syk, Spleen tyrosine kinase; Lyn, LYN, proto-oncogene; Src family tyrosine kinase; PKA, Protein kinase A; sCD40L, Soluble cluster of differentiation 40; CCL5, C-C motif chemokine ligand 5; IL-1β, Interleukin-1, beta; p50, Nuclear Factor Kappa B Subunit p50; p38, p38 mitogen activated protein kinase; TXB, Thromboxane B; LAT, Linker for activation of T cells; Fyn, Proto-oncogene tyrosine-protein kinase Fyn; FGA, fibrinogen alpha chain; FGB, fibrinogen beta chain; FGG, fibrinogen gamma chain; F7, Coagulation Factor VII; APTT, activated partial thromboplastin time; PT, prothrombin time; TT, thrombin time; EPCs, Endothelial progenitor cells.

#### 2.3.2 Capsaicin

Capsaicin, also known as capsaicin, is a vanillylamide alkaloid derived from plants of the genus *Capsicum*, with antioxidant, antitumor, antiulcer and analgesic effects (Wang Y. et al., 2021; Zhang W. et al., 2024). Capsaicin has been found to inhibit the formation of collagen fibers, which may inhibit blood clot formation (Perumal et al., 2015). Capsaicin was found to reduce mortality in acute pulmonary thromboembolism models prepared using ADP-induced mice and thromboembolism models prepared using collagen and sodium AA. Moreover, *in vitro* experiments revealed that capsaicin significantly inhibited platelet aggregation. In addition, capsaicin inhibited thrombosis more strongly than aspirin and indomethacin (Wang et al., 1985). In short, capsaicin exhibited antithrombotic effects by inhibiting platelet aggregation and collagen fibers.

#### 2.3.3 Ligustrazine

Ligustrazine, also known as tetramethylpyrazine, is an alkaloid extracted from the Chinese medicine *Ligusticum chuanxiong* with pharmacological activities such as anti-inflammatory, antioxidant and anti-apoptotic properties (Lin et al., 2022). It has been shown that tetramethylpyrazine inhibited ADP-induced platelet aggregation, reduced P-selectin secretion and glycoprotein (GP) IIb/IIIa expression, and decreased the release of inflammatory mediators soluble cluster of differentiation 40 (sCD40L) and Interleukin-1 beta (IL-1β) by stimulating the production of cyclic adenosine monophosphate (cAMP), the phosphorylation of vasodilator-stimulated phosphorylated protein ser157 (VASP^ser157^), and the dephosphorylation of Akt, indicating that tetramethylpyrazine has antiplatelet and thrombotic diseases prevention effects (Guan et al., 2022). Tetramethylpyrazine was found to protect endothelial cells and inhibit adrenaline-induced thrombosis in zebrafish by activating the MAPK signaling pathway, attenuating oxidative stress, and resisting cell apoptosis (Zhang Y. et al., 2022). In brief, ligustrazine prevented thrombosis by inhibiting platelet aggregation and protecting endothelial cells.

### 2.4 Terpenoids

Terpenoids are isoprene-based natural products with fundamental roles in the metabolism of all organisms, including andrographolide, paeoniflorin, tanshinone and glycyrrhizin (Bergman et al., 2019).

#### 2.4.1 Andrographolide

Andrographolide is a diterpenoid extracted from *Andrographis paniculata* with pharmacological activities such as anti-inflammatory, antioxidant, anticancer, antimicrobial, and antihyperglycemia (Li et al., 2022; Zeng et al., 2022). Andrographolide was found to inhibit p50 and TF expression in a mouse deep vein thrombosis model prepared by complete IVC ligation, thereby inhibiting thrombosis (as shown in Table 4) (Li et al., 2009). It was also shown that andrographolide inhibited thrombin-induced platelet aggregation by inhibiting the ERK1/2 pathway in a concentration- and time-dependent manner *in vitro*, indicating that andrographolide has antiplatelet effects and may prevent thrombosis-related diseases (Thisoda et al., 2006).

#### 2.4.2 Paeoniflorin

Paeoniflorin is a water-soluble monoterpene glucoside extracted from the root of the plant *Paeonia lactiflora*, with anti-inflammatory and immunomodulatory effects (Zhang and Wei, 2020; Zhang X. X. et al., 2022). It was shown that paeoniflorin inhibited shear stress-induced platelet aggregation and FeCl_3_-induced carotid artery thrombosis by inhibiting vascular hemophilic factor (vWF)-platelet glycoprotein Ib (GPIb) interaction (Ngo et al., 2019). It has also been shown that in a deep vein thrombosis model prepared by incomplete IVC ligation in rats, an aqueous extract of *Paeonia lactiflora*, the main component of which is paeoniflorin, inhibited inflammation by suppressing glycogen synthase kinase 3 beta (GSK3β) activity, thereby inhibiting thrombosis (as shown in Table 4) (Lu et al., 2021). In brief, paeoniflorin exerted antithrombotic effects through antiplatelet and anti-inflammatory pathways.

#### 2.4.3 Tanshinone

Tanshinone is a lipophilic diterpene isolated from the rhizome of *Salvia miltiorrhiza*, which possesses a variety of pharmacological activities such as anti-inflammatory, antioxidant, and antiapoptosis (Subedi and Gaire, 2021). Tanshinone IIA was shown to prevent thrombosis by inhibiting platelet activation through downregulation of CD36 and MKK4/JNK2 signaling pathways (Wang et al., 2020). It was found that tanshinone inhibited thrombin-induced platelet activation, aggregation, and adhesion through downregulation of the Akt/ERK and cSrc/RhoA pathways *in vitro.* Moreover, it was found that tanshinone inhibited the carotid thrombosis induced by FeCl_3_ *in vivo*, indicating that tanshinone had antithrombotic activity (Zhang Y. et al., 2024).

#### 2.4.4 Glycyrrhizin

Glycyrrhizin is an oleanane-type pentacyclic triterpenoid compound extracted from the roots and stems of *Glycyrrhiza uralensis Fisch*, a leguminous plant, which is known for its antiviral, immunomodulatory, and hepatoprotective effects (Mou et al., 2024). A previous study found that glycyrrhizin could exert antithrombotic effects by inhibiting the activity of thrombin (Mendes-Silva et al., 2003). In another study, glycyrrhizin was found to inhibit venous thrombosis by inhibiting the adhesion of neutrophils to the venous endothelium in a rat deep vein thrombosis model prepared by ligating the IVC (as shown in Table 4) (Nakata et al., 2008).

### 2.5 Saponin

Saponins are compounds consisting of glycosides (ligands) and sugar chains linked by glycosidic bonds, and are natural products from a wide range of sources, with pharmacological activities such as anti-inflammatory, immunosuppressive, and antithrombotic (Reichert et al., 2019).

#### 2.5.1 Ginsenosides

**FIGURE 2:**
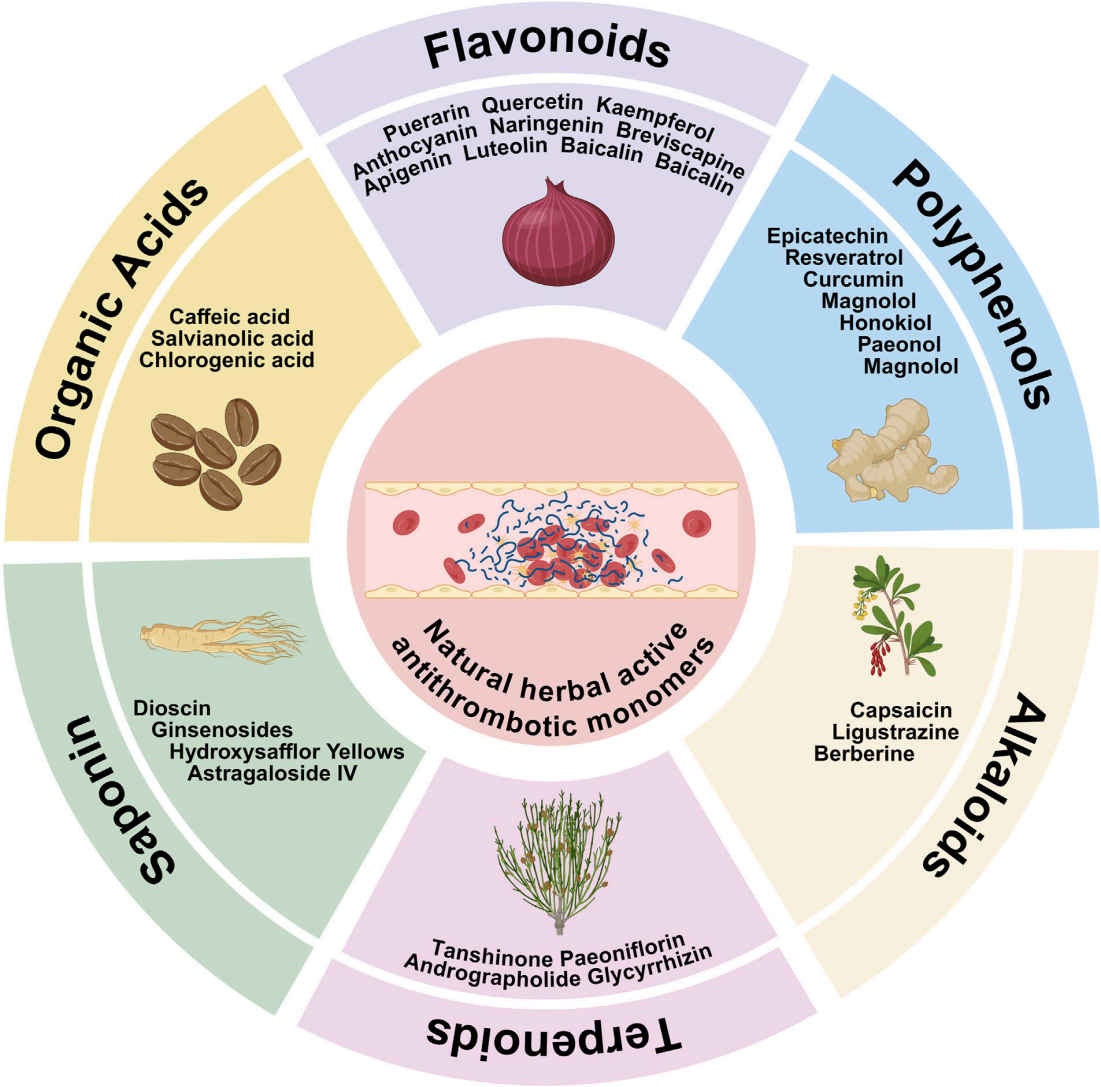
Natural active herbal antithrombotic monomers.

Ginsenoside is the main active ingredient of *Panax ginseng*, *Panax quinquefolius* and *Panax notoginseng*, belonging to the triterpenoid saponin class of compounds, with more than 40 components, including Rg (protopanaxatriol (PPT)-type within Rg-series), Rb (protopanaxadiol (PPD)-type, Rb-series), and Rh (low-glycosylated Rb-metabolite), according to their aglycone skeleton and sugar number (Figure 2) (Liu et al., 2021; Miao et al., 2022). It was shown that ginsenoside-Rk1 dose-dependently inhibited collagen and thrombin-induced platelet aggregation, Ca^2+^ release from the endoplasmic reticulum, granule release, and integrin α_IIb_β_3_ without any cytotoxic effects (Shin et al., 2021). It has also been shown that ginsenoside-Rp3 suppressed collagen, ADP, and thrombin-induced platelet aggregation through inhibition of the MAPK pathway and cyclic nucleotide signaling, and that ginsenoside-Rp3 also inhibited thrombus formation in an acute pulmonary thromboembolism model (Irfan et al., 2018). In addition, ginsenoside-RP1 was found to repress collagen, thrombin or ADP-induced platelet activation and aggregation and thrombosis by inhibiting GPVI, tyrosine phosphorylation and MAPK signaling pathways (Endale et al., 2012). It has also been found that ginsenoside-Rk3 inhibited collagen-induced platelet aggregation and exerted antithrombotic effects by up-regulating cAMP and PI3K/MAPK pathways (as shown in Table 4) (Kwon et al., 2023). These findings underscored multifaceted antithrombotic mechanisms and therapeutic potential of ginsenosides. Hereafter, ginsenosides are grouped as Rb-series (PPD-type, e.g., Rb_1_), Rg-series (PPT-type, e.g., Rg_1_), and Rh-series (deglycosylated metabolites, e.g., Rh_2_).

#### 2.5.2 Astragaloside IV

Astragaloside IV is a cyclic terpene triterpenoid saponin extracted from the Chinese herb *Astragalus*, which possesses anti-inflammatory, antioxidant and anti-apoptotic properties (Zhang et al., 2020). It was shown that astragaloside IV inhibited deep vein thrombosis by inhibiting PI3K/AKT signaling, suppressing inflammation, and promoting neovascularization in a deep vein thrombosis model prepared by incompletely IVC ligation in rats, indicating that astragaloside IV had antithrombotic activity (as shown in Table 4) (Lyu et al., 2024).

#### 2.5.3 Dioscin

Dioscin is a saponin compound extracted from the plant Dioscoreaceae with cardiovascular protective effects (Li et al., 2021). It was found that a mixture of total steroidal saponins (one of the main components is diosgenin) extracted from the rhizomes of the plant *Dioscorea* spp. exerted an antithrombotic effect by inhibiting platelet aggregation in a deep vein thrombosis model prepared by IVC ligating in rats, indicating that dioscin had antithrombotic activity (Li et al., 2010).

#### 2.5.4 Hydroxysafflor yellows

Hydroxysafflor yellows are mainly derived from the plant safflower (*Carthamus tinctorius Safflower*) and belongs to the monochalcone glycosides. It was found that hydroxy saffron yellow inhibited phenylhydrazine-induced thrombosis in zebrafish by enhancing blood circulation and toxic excretion, indicating that hydroxysafflor had antithrombotic activity (as shown in Table 4) (Wang L. W. et al., 2021).

### 2.6 Organic acids

#### 2.6.1 Caffeic acid

Caffeic acid is a common phenolic acid found in coffee and many fruits and vegetables, known for its antioxidant properties (Calabrese et al., 2024). It was shown that caffeic acid not only inhibited photochemical damage-induced thrombosis in mouse cerebral arteries, but also reduced platelet deposition and prolonged vascular occlusion, while caffeic acid also inhibited ADP induced cerebral venous thrombosis and platelet adherence in mice, and reduced the thrombus/vein area ratio. In addition, *in vitro* experiments in this study showed that caffeic acid also inhibited ADP-induced platelet aggregation, p-selectin expression, ATP release, Ca^2+^ mobilization, and integrin α_IIb_β_3_ activation, and reduced p38, ERK, and JNK activation, and increased cAMP levels, thereby inhibiting thrombosis (as shown in Table 4) (Lu et al., 2015). It has also been shown that caffeic acid inhibited collagen-induced platelet aggregation, thromboxane A_2_ (TXA_2_) production, and Ca^2+^ mobilization in a concentration-dependent manner, increased cAMP and cGMP levels, and increased the phosphorylation of inositol 1,4,5-trisphosphate receptor (IP_3_R), thereby preventing collagen-induced platelet aggregation and reducing the risk of thrombosis (Lee et al., 2014). Recent studies have shown that caffeic acid inhibited thrombin-induced clot retraction and decreased cAMP levels in platelets, inhibited phosphorylation of Akt and ERK, as well as enhanced phosphorylation of VASP, reducing intracellular Ca^2+^ mobilization, ATP release, p-selectin expression, and binding of fibrinogen to integrin α_IIb_β_3_, thereby attenuating platelet activation and inhibiting thrombosis (Nam et al., 2020). In short, caffeic acid inhibited thrombosis by blocking platelet activation and multiple signaling pathways.

#### 2.6.2 Salvianolic acid

Salvianolic acid is a water-soluble, weakly acidic drug extracted from the roots and rhizomes of *Salvia miltiorrhiza Bunge*, family Labiatae, with anti-inflammatory, antioxidant and anti-tumor effects (He et al., 2023). It has been shown that salvinorin A dose-dependently inhibited ADP, thrombin, collagen-induced platelet aggregation, P-selectin expression, and fibrinogen binding through inhibition of the PI3K pathway, and also inhibited photochemically induced carotid thrombosis (Huang et al., 2010). Salvianolic acid B was found to dose-dependently inhibit thrombin, ADP, and collagen-induced platelet activation and aggregation, and to reduce FeCl_3_-induced carotid artery thrombosis and photochemical injury-induced thrombosis of small arteries of the raphe in mice (as shown in Table 4) (Neves et al., 2024). In, brief, salvianolic acid inhibited platelet aggregation and thrombosis in multiple models.

#### 2.6.3 Chlorogenic acid

Chlorogenic acid is widely found in plant foods and is known for its antioxidant, anti-inflammatory, antibacterial, and antiviral activities (Singh et al., 2023). It was shown that chlorogenic acid inhibited ADP, collagen, and AA-induced platelet aggregation and adhesion in a dose-dependent manner through modulation of the A_2_A receptor, adenylate cyclase, and cAMP/PKA signaling pathways, and inhibited the expression of inflammatory mediators (sCD40L, CCL5, and IL-1β) by inhibiting thrombus in photochemically injured mouse mesenteric arteries (Fuentes et al., 2014). A mouse model of acute thromboembolism induced by collagen and epinephrine revealed that chlorogenic acid inhibited thrombosis by inhibiting the activities of procoagulant protease, thrombin, activated FXa, and activated factor XIII (FXIIIa) and delaying APTT, PT, and thrombin time (as shown in Table 4) (Choi and Kim, 2017).

### 2.7 Synthesis of mechanisms: novel targets and synergistic effects

A key advantage of the herbal monomers discussed is their ability to modulate a wide array of targets, encompassing both established and novel antithrombotic pathways. Many compounds, such as baicalin and kaempferol, inhibit thrombin and Factor Xa, which are the targets of conventional DOACs, suggesting they can improve upon existing mechanisms (Choi et al., 2015b; Lee et al., 2015). Similarly, compounds like caffeic acid and berberine interfere with platelet activation pathways involving PI3K, which are also targeted by synthetic drugs (Lu et al., 2015; Wang C. et al., 2021).

More importantly, this review highlights that natural products often engage novel or upstream targets not typically addressed by current pharmaceuticals. For instance, puerarin, curcumin, and resveratrol have been shown to suppress thrombosis by inhibiting inflammation through the NF-κB and NLRP3 signaling pathways (Deng et al., 2017; Liang et al., 2021; Fei et al., 2022). This approach targets the root causes of thrombus formation, representing a potentially more holistic treatment strategy. Furthermore, several compounds promote thrombus resolution, a mechanism distinct from prevention. Paeonol and epigallocatechin-3-gallate, for example, facilitate neovascularization and endothelial progenitor cell (EPC) function to actively resolve existing clots (Ye et al., 2016; Li et al., 2025).

The therapeutic potential of these natural monomers likely stems from their synergistic, multi-target action. A single compound can simultaneously inhibit platelet aggregation, reduce coagulation, suppress inflammation, and promote clot resolution, as summarized. This pleiotropic activity could lead to a more effective antithrombotic outcome with a potentially lower risk of bleeding compared to single-target synthetic drugs, making them highly attractive candidates for the development of the next-generation of antithrombotic therapies.

## 3 Conclusion and future perspectives

Due to the rising incidence of thromboembolic diseases and the defect of existing treatments, seeking new treatments and drugs is urgent and necessary. Natural plant herbs and their active monomers have shown unique advantages in the prevention and treatment of thromboembolic diseases. The antithrombotic mechanism of natural herbal monomers mainly focuses on the inhibition of platelet aggregation or thrombin activity, inhibition of procoagulant protease, prothrombin, activated FXa, and activated FXIIIa activities, and prolonged APTT, PT, and PT. In addition, some active ingredients can also inhibit inflammatory factors in thrombus, suppress the adhesion between leukocytes, platelets and endothelial cells, and promote neovascularization in thrombus to promote thrombus resolution, and have a multi-target synergistic effect. This review systematically summarizes the antithrombotic potential of various active monomers from Chinese herbs demonstrated in preclinical models. The vast majority of these findings have not yet entered clinical trials, highlighting the core purpose of this study: to comprehensively evaluate and screen promising natural drug candidates before initiating expensive and complex clinical research.

However, despite the significant antithrombotic potential of natural herbal monomers, most of the current studies have been limited to *ex vivo* experiments and rat or mouse models, with a lack of clinical trials, and still face many challenges. In addition, the low activity and poor bioavailability of the monomeric portion of natural active herbs require optimization of the structure or modification of the dosage form to improve activity and bioavailability. Existing antithrombotic drugs, such as warfarin and novel oral anticoagulants, are effective but still face challenges of bleeding risk and narrow therapeutic windows. The herbal monomers revealed in this review act through multiple targets and pathways—such as inhibiting platelet activation, regulating the coagulation cascade, and exerting anti-inflammatory and antioxidant effects. This synergistic action could translate into a lower risk of bleeding and superior therapeutic outcomes, offering new approaches to overcome current clinical dilemmas (Atanasov et al., 2021).

Nanoparticles and liposomes can be developed to enhance the bioavailability of natural herbal monomers in the future. Combining artificial intelligence large models, single-cell omics, molecular docking and other technologies for target prediction, to clarify the new mechanisms by which natural herbal monomers and others regulate thrombus. Adopt a prevention-first treatment strategy, expand the natural herbal monomers obtained from food, and reduce the incidence of thrombus and protect the cardiovascular system through early intervention. The ultimate goal of this review is to pave the way for future clinical research. We propose that the next step should be to select one or two of the most promising compounds based on the evidence presented here (e.g., resveratrol, salvianolic acid B), conduct more standardized animal model studies, and ultimately design rigorous, scientific, small-scale Phase I or II clinical trials to evaluate their safety, tolerability, and preliminary efficacy in humans. At the same time, expanding the scope of research from treatment to prevention—especially for active ingredients derived from everyday foods (such as quercetin and anthocyanins)—could offer safe and convenient new strategies for the primary prevention of thrombotic diseases through dietary supplements or functional foods.

In conclusion, this review is not just a compilation of past research but also a roadmap for future studies. We have clearly identified the hurdles to be overcome in moving from preclinical evidence to clinical application and have proposed specific strategies to address them. We believe this work provides a valuable reference for the development of safer, more effective, next-generation antithrombotic drugs derived from nature and holds significant translational value.

## References

[B1] Abd El-Hack M. E. El-Saadony M. T. Swelum A. A. Arif M. Abo Ghanima M. M. Shukry M. (2021). Curcumin, the active substance of turmeric: its effects on health and ways to improve its bioavailability. J. Sci. Food Agric. 101 (14), 5747–5762. 10.1002/jsfa.11372 34143894

[B2] Aboonabi A. Meyer R. R. Gaiz A. Singh I. (2020). Anthocyanins in berries exhibited anti-atherogenicity and antiplatelet activities in a metabolic syndrome population. Nutr. Res. 76, 82–93. 10.1016/j.nutres.2020.02.011 32217379

[B3] Ashorobi D. Ameer M. A. Fernandez R. (2025). “Thrombosis,” in StatPearls. (Treasure Island, FL: StatPearls Publishing). 30860701

[B4] Atanasov A. G. Zotchev S. B. Dirsch V. M. International Natural Product Sciences T. Supuran C. T. (2021). Natural products in drug discovery: advances and opportunities. Nat. Rev. Drug Discov. 20 (3), 200–216. 10.1038/s41573-020-00114-z 33510482 PMC7841765

[B5] Avula B. Katragunta K. Osman A. G. Ali Z. John Adams S. Chittiboyina A. G. (2023). Advances in the chemistry, analysis and adulteration of anthocyanin rich-berries and fruits: 2000-2022. Molecules 28 (2), 560. 10.3390/molecules28020560 36677615 PMC9865467

[B6] Bergman M. E. Davis B. Phillips M. A. (2019). Medically useful plant terpenoids: biosynthesis, occurrence, and mechanism of action. Molecules 24 (21), 3961. 10.3390/molecules24213961 31683764 PMC6864776

[B7] Breuss J. M. Atanasov A. G. Uhrin P. (2019). Resveratrol and its effects on the vascular system. Int. J. Mol. Sci. 20 (7), 1523. 10.3390/ijms20071523 30934670 PMC6479680

[B8] Calabrese E. J. Pressman P. Hayes A. W. Baldwin L. Agathokleous E. Dhawan G. (2024). Caffeic acid: numerous chemoprotective effects are mediated *via* hormesis. J. Diet. Suppl. 21 (6), 842–867. 10.1080/19390211.2024.2410776 39363555

[B9] Chen S. Wang X. Cheng Y. Gao H. Chen X. (2023). A review of classification, biosynthesis, biological activities and potential applications of flavonoids. Molecules 28 (13), 4982. 10.3390/molecules28134982 37446644 PMC10343696

[B10] Choi J. H. Kim S. (2017). Investigation of the anticoagulant and antithrombotic effects of chlorogenic acid. J. Biochem. Mol. Toxicol. 31 (3), e21865. 10.1002/jbt.21865 27704645

[B11] Choi J. H. Kim Y. S. Shin C. H. Lee H. J. Kim S. (2015a). Antithrombotic activities of luteolin *in vitro* and *in vivo* . J. Biochem. Mol. Toxicol. 29 (12), 552–558. 10.1002/jbt.21726 26184785

[B12] Choi J. H. Park S. E. Kim S. J. Kim S. (2015b). Kaempferol inhibits thrombosis and platelet activation. Biochimie 115, 177–186. 10.1016/j.biochi.2015.06.001 26073152

[B13] Choi J. H. Kim K. J. Kim S. (2016). Comparative effect of quercetin and Quercetin-3-O-beta-d-Glucoside on fibrin polymers, blood clots, and in rodent models. J. Biochem. Mol. Toxicol. 30 (11), 548–558. 10.1002/jbt.21822 27271803

[B14] Choo M. K. Park E. K. Yoon H. K. Kim D. H. (2002). Antithrombotic and antiallergic activities of daidzein, a metabolite of puerarin and daidzin produced by human intestinal microflora. Biol. Pharm. Bull. 25 (10), 1328–1332. 10.1248/bpb.25.1328 12392089

[B15] Deng H. F. Wang X. L. Sun H. Xiao X. Z. (2017). Puerarin inhibits expression of tissue factor induced by oxidative low-density lipoprotein through activating the PI3K/Akt/eNOS pathway and inhibiting activation of ERK1/2 and NF-κB. Life Sci. 191, 115–121. 10.1016/j.lfs.2017.10.018 29037842

[B16] Dohare P. Garg P. Jain V. Nath C. Ray M. (2008). Dose dependence and therapeutic window for the neuroprotective effects of curcumin in thromboembolic model of rat. Behav. Brain Res. 193 (2), 289–297. 10.1016/j.bbr.2008.06.012 18611416

[B17] Endale M. Lee W. M. Kamruzzaman S. M. Kim S. D. Park J. Y. Park M. H. (2012). Ginsenoside-Rp1 inhibits platelet activation and thrombus formation *via* impaired glycoprotein VI signalling pathway, tyrosine phosphorylation and MAPK activation. Br. J. Pharmacol. 167 (1), 109–127. 10.1111/j.1476-5381.2012.01967.x 22471932 PMC3448917

[B18] Fei J. Qin X. Ma H. Zhang X. Wang H. Han J. (2022). Resveratrol ameliorates deep vein thrombosis-induced inflammatory response through inhibiting HIF-1α/NLRP3 pathway. Inflammation 45 (6), 2268–2279. 10.1007/s10753-022-01689-y 35655037

[B19] Fuentes E. Caballero J. Alarcon M. Rojas A. Palomo I. (2014). Chlorogenic acid inhibits human platelet activation and thrombus formation. PLoS One 9 (3), e90699. 10.1371/journal.pone.0090699 24598787 PMC3944540

[B20] Gailani D. Gruber A. (2024). Targeting factor XI and factor XIa to prevent thrombosis. Blood 143 (15), 1465–1475. 10.1182/blood.2023020722 38142404 PMC11033593

[B21] Gaiz A. Kundur A. R. Nikbakht E. Vugic L. Colson N. Shibeeb S. (2022). Anthocyanin supplementation alleviates antithrombotic risk by inhibiting platelet activity in humans. Altern. Ther. Health Med. 28 (2), 44–49. 33789251

[B22] Goldhaber S. Z. Magnuson E. A. Chinnakondepalli K. M. Cohen D. J. Vedantham S. (2021). Catheter-directed thrombolysis for deep vein thrombosis: 2021 update. Vasc. Med. 26 (6), 662–669. 10.1177/1358863X211042930 34606385 PMC9009765

[B23] Guan B. Gao J. Tan Y. Ma X. Shi D. (2022). Antiplatelet activity of tetramethylpyrazine *via* regulation of the P2Y12 receptor downstream signaling pathway. Evid. Based Complement. Altern. Med. 2022, 7941039. 10.1155/2022/7941039 35378909 PMC8976642

[B24] He G. Chen G. Liu W. Ye D. Liu X. Liang X. (2023). Salvianolic acid B: a review of pharmacological effects, safety, combination therapy, new dosage forms, and novel drug delivery routes. Pharmaceutics 15 (9), 2235. 10.3390/pharmaceutics15092235 37765204 PMC10538146

[B25] Helin T. A. Joutsi-Korhonen L. Asmundela H. Niemi M. Orpana A. Lassila R. (2019). Warfarin dose requirement in patients having severe thrombosis or thrombophilia. Br. J. Clin. Pharmacol. 85 (8), 1684–1691. 10.1111/bcp.13948 30933373 PMC6624394

[B26] Hu H. Zhang X. X. Wang Y. Y. Chen S. Z. (2005). Honokiol inhibits arterial thrombosis through endothelial cell protection and stimulation of prostacyclin. Acta Pharmacol. Sin. 26 (9), 1063–1068. 10.1111/j.1745-7254.2005.00164.x 16115372

[B27] Huang Z. S. Zeng C. L. Zhu L. J. Jiang L. Li N. Hu H. (2010). Salvianolic acid A inhibits platelet activation and arterial thrombosis *via* inhibition of phosphoinositide 3-kinase. J. Thromb. Haemost. 8 (6), 1383–1393. 10.1111/j.1538-7836.2010.03859.x 20345719

[B28] Huang M. Deng M. Nie W. Zou D. Wu H. Xu D. (2021). Naringenin inhibits platelet activation and arterial thrombosis through inhibition of phosphoinositide 3-Kinase and cyclic nucleotide signaling. Front. Pharmacol. 12, 722257. 10.3389/fphar.2021.722257 34475824 PMC8406801

[B29] Irfan M. Jeong D. Kwon H. W. Shin J. H. Park S. J. Kwak D. (2018). Ginsenoside-Rp3 inhibits platelet activation and thrombus formation by regulating MAPK and cyclic nucleotide signaling. Vasc. Pharmacol. 109, 45–55. 10.1016/j.vph.2018.06.002 29890296

[B30] Jiang Z. Cui X. Qu P. Shang C. Xiang M. Wang J. (2022). Roles and mechanisms of puerarin on cardiovascular disease:a review. Biomed. Pharmacother. 147, 112655. 10.1016/j.biopha.2022.112655 35066299

[B31] Keihanian F. Saeidinia A. Bagheri R. K. Johnston T. P. Sahebkar A. (2018). Curcumin, hemostasis, thrombosis, and coagulation. J. Cell Physiol. 233 (6), 4497–4511. 10.1002/jcp.26249 29052850

[B32] Khedr E. M. Abdelwarith A. Moussa G. Saber M. (2023). Recombinant tissue plasminogen activator (rTPA) management for first onset acute ischemic stroke with covid -19 and non-covid -19 patients. J. Stroke Cerebrovasc. Dis. 32 (4), 107031. 10.1016/j.jstrokecerebrovasdis.2023.107031 36701854 PMC9868389

[B33] Kim K. A. Choi S. Y. Kim R. (2021). Endovascular treatment for lower extremity deep vein thrombosis: an overview. Korean J. Radiol. 22 (6), 931–943. 10.3348/kjr.2020.0675 33660456 PMC8154777

[B34] Kwon H. W. Shin J. H. Rhee M. H. Park C. E. Lee D. H. (2023). Anti-thrombotic effects of ginsenoside Rk3 by regulating cAMP and PI3K/MAPK pathway on human platelets. J. Ginseng Res. 47 (6), 706–713. 10.1016/j.jgr.2023.04.006 38107398 PMC10721468

[B35] Lee D. H. Kim H. H. Cho H. J. Bae J. S. Yu Y. B. Park H. J. (2014). Antiplatelet effects of caffeic acid due to Ca(2+) mobilizationinhibition via cAMP-dependent inositol-1, 4, 5-trisphosphate receptor phosphorylation. J. Atheroscler. Thromb. 21 (1), 23–37. 10.5551/jat.18994 24088646

[B36] Lee W. Ku S. K. Bae J. S. (2015). Antiplatelet, anticoagulant, and profibrinolytic activities of baicalin. Arch. Pharm. Res. 38 (5), 893–903. 10.1007/s12272-014-0410-9 24849036

[B37] Lee T. Y. Chang C. C. Lu W. J. Yen T. L. Lin K. H. Geraldine P. (2017). Honokiol as a specific collagen receptor glycoprotein VI antagonist on human platelets: functional *ex vivo* and *in vivo* studies. Sci. Rep. 7, 40002. 10.1038/srep40002 28054640 PMC5213647

[B38] Li Y. D. Ye B. Q. Zheng S. X. Wang J. T. Wang J. G. Chen M. (2009). NF-kappaB transcription factor p50 critically regulates tissue factor in deep vein thrombosis. J. Biol. Chem. 284 (7), 4473–4483. 10.1074/jbc.M806010200 19095643 PMC2640971

[B39] Li H. Huang W. Wen Y. Gong G. Zhao Q. Yu G. (2010). Anti-thrombotic activity and chemical characterization of steroidal saponins from Dioscorea zingiberensis C.H. wright. Fitoterapia 81 (8), 1147–1156. 10.1016/j.fitote.2010.07.016 20659537

[B40] Li X. Liu S. Qu L. Chen Y. Yuan C. Qin A. (2021). Dioscin and diosgenin: insights into their potential protective effects in cardiac diseases. J. Ethnopharmacol. 274, 114018. 10.1016/j.jep.2021.114018 33716083

[B41] Li X. Yuan W. Wu J. Zhen J. Sun Q. Yu M. (2022). Andrographolide, a natural anti-inflammatory agent: an Update. Front. Pharmacol. 13, 920435. 10.3389/fphar.2022.920435 36238575 PMC9551308

[B42] Li D. Mao Y. Zhang X. Wang Y. Tang H. Huang H. (2025). Epigallocatechin-3-Gallate promotes recanalization in deep vein thrombosis by modulating endothelial progenitor cell ferroptosis through the Nrf2 pathway. Phytother. Res. 39 (3), 1632–1644. 10.1002/ptr.8457 39918021

[B43] Liang D. Wen Z. Han W. Li W. Pan L. Zhang R. (2021). Curcumin protects against inflammation and lung injury in rats with acute pulmonary embolism with the involvement of microRNA-21/PTEN/NF-κB axis. Mol. Cell Biochem. 476 (7), 2823–2835. 10.1007/s11010-021-04127-z 33730297

[B44] Lin J. Wang Q. Zhou S. Xu S. Yao K. (2022). Tetramethylpyrazine: a review on its mechanisms and functions. Biomed. Pharmacother. 150, 113005. 10.1016/j.biopha.2022.113005 35483189

[B45] Lin S. Ma H. Zhang S. Fan W. Shen C. Chen J. (2024). The combination of paeonol, diosmetin-7-O-beta-D-glucopyranoside, and 5-hydroxymethylfurfural from trichosanthis pericarpium alleviates arachidonic acid-induced thrombosis in a zebrafish model. Front. Pharmacol. 15, 1332468. 10.3389/fphar.2024.1332468 38487165 PMC10937350

[B46] Liu M. Y. Liu F. Gao Y. L. Yin J. N. Yan W. Q. Liu J. G. (2021). Pharmacological activities of ginsenoside Rg5 (review). Exp. Ther. Med. 22 (2), 840. 10.3892/etm.2021.10272 34149886 PMC8210315

[B47] Liu S. Zheng X. Luo Z. Tang C. Hu Y. Peng Q. (2024). The synthesis and bioactivity of apigenin derivatives. Fitoterapia 179, 106228. 10.1016/j.fitote.2024.106228 39332505

[B48] Lu Y. Li Q. Liu Y. Y. Sun K. Fan J. Y. Wang C. S. (2015). Inhibitory effect of caffeic acid on ADP-Induced thrombus formation and platelet activation involves mitogen-activated protein kinases. Sci. Rep. 5, 13824. 10.1038/srep13824 26345207 PMC4561902

[B49] Lu Z. Wang S. Zhu X. Yuan X. Zhan Y. Li Y. (2019). Resveratrol induces endothelial progenitor cells angiogenesis *via* MiR-542-3p by targeting Angiopoietin-2 and involves in recanalization of venous thrombosis. Med. Sci. Monit. 25, 7675–7683. 10.12659/MSM.917013 31606730 PMC6807528

[B50] Lu Z. Ye Y. Liu Y. Yang X. Ding Q. Wang Y. (2021). Aqueous extract of paeoniae Radix rubra prevents deep vein thrombosis by ameliorating inflammation through inhibiting GSK3β activity. Phytomedicine 92, 153767. 10.1016/j.phymed.2021.153767 34597905

[B51] Lyu X. Yi Z. He Y. Zhang C. Zhu P. Liu C. (2024). Astragaloside IV induces endothelial progenitor cell angiogenesis in deep venous thrombosis through inactivation of PI3K/AKT signaling. Histol. Histopathol. 39 (9), 1149–1157. 10.14670/HH-18-704 38275076

[B52] Manjunath S. H. Thimmulappa R. K. (2022). Antiviral, immunomodulatory, and anticoagulant effects of quercetin and its derivatives: potential role in prevention and management of COVID-19. J. Pharm. Anal. 12 (1), 29–34. 10.1016/j.jpha.2021.09.009 34567823 PMC8450231

[B53] Mattioli R. Francioso A. Mosca L. Silva P. (2020). Anthocyanins: a comprehensive review of their chemical properties and health effects on cardiovascular and neurodegenerative diseases. Molecules 25 (17), 3809. 10.3390/molecules25173809 32825684 PMC7504512

[B54] Mayanglambam A. Dangelmaier C. A. Thomas D. Damodar Reddy C. Daniel J. L. Kunapuli S. P. (2010). Curcumin inhibits GPVI-Mediated platelet activation by interfering with the kinase activity of Syk and the subsequent activation of PLCgamma2. Platelets 21 (3), 211–220. 10.3109/09537100903528269 20158382

[B55] Mendes-Silva W. Assafim M. Ruta B. Monteiro R. Q. Guimaraes J. A. Zingali R. B. (2003). Antithrombotic effect of glycyrrhizin, a plant-derived thrombin inhibitor. Thromb. Res. 112 (1-2), 93–98. 10.1016/j.thromres.2003.10.014 15013279

[B56] Miao L. Yang Y. Li Z. Fang Z. Zhang Y. Han C. C. (2022). Ginsenoside Rb2: a review of pharmacokinetics and pharmacological effects. J. Ginseng Res. 46 (2), 206–213. 10.1016/j.jgr.2021.11.007 35509822 PMC9058830

[B57] Montalvan Ayala V. Rojas Cheje Z. Aldave Salazar R. (2022). Controversies in cerebrovascular disease: high or low doses of recombinant tissue plasminogen activator to treat acute stroke? A literature review. Neurol. Engl. Ed. 37 (2), 130–135. 10.1016/j.nrl.2018.04.003 35279226

[B58] Mosawy S. Jackson D. E. Woodman O. L. Linden M. D. (2013). Treatment with quercetin and 3',4'-dihydroxyflavonol inhibits platelet function and reduces thrombus formation *in vivo* . J. Thromb. Thrombolysis 36 (1), 50–57. 10.1007/s11239-012-0827-2 23070586

[B59] Motallebi M. Bhia M. Rajani H. F. Bhia I. Tabarraei H. Mohammadkhani N. (2022). Naringenin: a potential flavonoid phytochemical for cancer therapy. Life Sci. 305, 120752. 10.1016/j.lfs.2022.120752 35779626

[B60] Mou Y. Liao W. Li Y. Wan L. Liu J. Luo X. (2024). Glycyrrhizin and the related preparations: an inspiring resource for the treatment of liver diseases. Am. J. Chin. Med. 52 (2), 315–354. 10.1142/S0192415X24500149 38553799

[B61] Mu K. Liu Y. Liu G. Ran F. Zhou L. Wu Y. (2023). A review of hemostatic chemical components and their mechanisms in traditional Chinese medicine and ethnic medicine. J. Ethnopharmacol. 307, 116200. 10.1016/j.jep.2023.116200 36739925

[B62] Nakata N. Kira Y. Yabunaka Y. Takaoka K. (2008). Prevention of venous thrombosis by preoperative glycyrrhizin infusion in a rat model. J. Orthop. Sci. 13 (5), 456–462. 10.1007/s00776-008-1259-x 18843461

[B63] Nam G. S. Park H. J. Nam K. S. (2020). The antithrombotic effect of caffeic acid is associated with a cAMP-dependent pathway and clot retraction in human platelets. Thromb. Res. 195, 87–94. 10.1016/j.thromres.2020.07.024 32682003

[B64] Navarro-Nunez L. Lozano M. L. Palomo M. Martinez C. Vicente V. Castillo J. (2008). Apigenin inhibits platelet adhesion and thrombus formation and synergizes with aspirin in the suppression of the arachidonic acid pathway. J. Agric. Food Chem. 56 (9), 2970–2976. 10.1021/jf0723209 18410117

[B65] Neves M. A. D. Ni T. T. Mackeigan D. T. Shoara A. A. Lei X. Slavkovic S. (2024). Salvianolic acid B inhibits thrombosis and directly blocks the thrombin catalytic site. Res. Pract. Thromb. Haemost. 8 (4), 102443. 10.1016/j.rpth.2024.102443 38993621 PMC11238050

[B66] Newman D. J. Cragg G. M. (2020). Natural products as sources of new drugs over the nearly four decades from 01/1981 to 09/2019. J. Nat. Prod. 83 (3), 770–803. 10.1021/acs.jnatprod.9b01285 32162523

[B67] Ngo T. Kim K. Bian Y. Noh H. Lim K. M. Chung J. H. (2019). Antithrombotic effects of paeoniflorin from Paeonia suffruticosa by selective inhibition on shear stress-induced Platelet aggregation. Int. J. Mol. Sci. 20 (20), 5040. 10.3390/ijms20205040 31614534 PMC6834133

[B68] Oh T. W. Do H. J. Jeon J. H. Kim K. (2021). Quercitrin inhibits platelet activation in arterial thrombosis. Phytomedicine 80, 153363. 10.1016/j.phymed.2020.153363 33070081

[B69] Ortel T. L. Neumann I. Ageno W. Beyth R. Clark N. P. Cuker A. (2020). American society of Hematology 2020 guidelines for management of venous thromboembolism: treatment of deep vein thrombosis and pulmonary embolism. Blood Adv. 4 (19), 4693–4738. 10.1182/bloodadvances.2020001830 33007077 PMC7556153

[B70] Periferakis A. Periferakis K. Badarau I. A. Petran E. M. Popa D. C. Caruntu A. (2022). Kaempferol: antimicrobial properties, sources, clinical, and traditional applications. Int. J. Mol. Sci. 23 (23), 15054. 10.3390/ijms232315054 36499380 PMC9740324

[B71] Perumal S. Dubey K. Badhwar R. George K. J. Sharma R. K. Bagler G. (2015). Capsaicin inhibits collagen fibril formation and increases the stability of collagen fibers. Eur. Biophys. J. 44 (1-2), 69–76. 10.1007/s00249-014-1002-9 25528374

[B72] Qi W. Qi W. Xiong D. Long M. (2022). Quercetin: its antioxidant mechanism, antibacterial properties and potential application in prevention and control of toxipathy. Molecules 27 (19), 6545. 10.3390/molecules27196545 36235082 PMC9571766

[B73] Reichert C. L. Salminen H. Weiss J. (2019). Quillaja saponin characteristics and functional properties. Annu. Rev. Food Sci. Technol. 10, 43–73. 10.1146/annurev-food-032818-122010 30664381

[B74] Sagris M. Tzoumas A. Kokkinidis D. G. Korosoglou G. Lichtenberg M. Tzavellas G. (2022). Invasive and pharmacological treatment of deep vein thrombosis: a scoping review. Curr. Pharm. Des. 28 (10), 778–786. 10.2174/1381612828666220418084339 35440298

[B75] Shih C. Y. Chou T. C. (2012). The antiplatelet activity of magnolol is mediated by PPAR-β/γ. Biochem. Pharmacol. 84 (6), 793–803. 10.1016/j.bcp.2012.06.022 22750553

[B76] Shin J. H. Kwon H. W. Irfan M. Rhee M. H. Lee D. H. (2021). Ginsenoside Rk1 suppresses platelet mediated thrombus formation by downregulation of granule release and α_IIb_β_3_ activation. J. Ginseng Res. 45 (4), 490–497. 10.1016/j.jgr.2020.11.001 34295209 PMC8282495

[B77] Si H. Lai C. Q. Liu D. (2021). Dietary epicatechin, A novel anti-aging bioactive small molecule. Curr. Med. Chem. 28 (1), 3–18. 10.2174/0929867327666191230104958 31886745

[B78] Sinegre T. Teissandier D. Milenkovic D. Morand C. Lebreton A. (2019). Epicatechin influences primary hemostasis, coagulation and fibrinolysis. Food Funct. 10 (11), 7291–7298. 10.1039/c9fo00816k 31621731

[B79] Singh P. Arif Y. Bajguz A. Hayat S. (2021). The role of quercetin in plants. Plant Physiol. Biochem. 166, 10–19. 10.1016/j.plaphy.2021.05.023 34087741

[B80] Singh A. K. Singla R. K. Pandey A. K. (2023). Chlorogenic acid: a dietary phenolic acid with promising pharmacotherapeutic potential. Curr. Med. Chem. 30 (34), 3905–3926. 10.2174/0929867329666220816154634 35975861

[B81] Song D. Hao J. Fan D. (2020). Biological properties and clinical applications of berberine. Front. Med. 14 (5), 564–582. 10.1007/s11684-019-0724-6 32335802

[B82] Stainer A. R. Sasikumar P. Bye A. P. Unsworth A. J. Holbrook L. M. Tindall M. (2019). The metabolites of the dietary flavonoid Quercetin possess potent antithrombotic activity, and interact with aspirin to enhance antiplatelet effects. TH Open 3 (3), e244–e258. 10.1055/s-0039-1694028 31367693 PMC6667742

[B83] Subedi L. Gaire B. P. (2021). Tanshinone IIA: a phytochemical as a promising drug candidate for neurodegenerative diseases. Pharmacol. Res. 169, 105661. 10.1016/j.phrs.2021.105661 33971269

[B84] Sun Z. Zhao T. Bai X. Li H. Gao J. Hao Y. (2024). Berberine targets PKM2 to activate the t-PA-Induced fibrinolytic System and improves thrombosis. Pharm. (Basel) 17 (9), 1219. 10.3390/ph17091219 39338381 PMC11434879

[B85] Teng C. M. Chen C. C. Ko F. N. Lee L. G. Huang T. F. Chen Y. P. (1988). Two antiplatelet agents from Magnolia officinalis. Thromb. Res. 50 (6), 757–765. 10.1016/0049-3848(88)90336-2 3413728

[B86] Thisoda P. Rangkadilok N. Pholphana N. Worasuttayangkurn L. Ruchirawat S. Satayavivad J. (2006). Inhibitory effect of Andrographis paniculata extract and its active diterpenoids on platelet aggregation. Eur. J. Pharmacol. 553 (1-3), 39–45. 10.1016/j.ejphar.2006.09.052 17081514

[B87] Wang J. P. Hsu M. F. Hsu T. P. Teng C. M. (1985). Antihemostatic and antithrombotic effects of capsaicin in comparison with aspirin and indomethacin. Thromb. Res. 37 (6), 669–679. 10.1016/0049-3848(85)90196-3 3992533

[B88] Wang Y. Yang X. Liu H. Tang X. (2003). Study on effects of Erigeron breviscapus extract on anticoagulation. Zhong Yao Cai 26 (9), 656–658. 14692325

[B89] Wang S. B. Jang J. Y. Chae Y. H. Min J. H. Baek J. Y. Kim M. (2015). Kaempferol suppresses collagen-induced platelet activation by inhibiting NADPH oxidase and protecting SHP-2 from oxidative inactivation. Free Radic. Biol. Med. 83, 41–53. 10.1016/j.freeradbiomed.2015.01.018 25645952

[B90] Wang H. Zhong L. Mi S. Song N. Zhang W. Zhong M. (2020). Tanshinone IIA prevents platelet activation and down-regulates CD36 and MKK4/JNK2 signaling pathway. BMC Cardiovasc. Disord. 20 (1), 81. 10.1186/s12872-019-01289-z 32059638 PMC7023810

[B91] Wang C. Cheng Y. Zhang Y. Jin H. Zuo Z. Wang A. (2021a). Berberine and its main metabolite berberrubine inhibit Platelet activation through suppressing the class I PI3Kβ/Rasa3/Rap1 pathway. Front. Pharmacol. 12, 734603. 10.3389/fphar.2021.734603 34690771 PMC8531212

[B92] Wang L. W. Cui X. Y. He J. F. Duan S. Liu C. R. Shan C. B. (2021b). Hydroxysafflor yellows alleviate thrombosis and acetaminophen-induced toxicity *in vivo* by enhancing blood circulation and poison excretion. Phytomedicine 87, 153579. 10.1016/j.phymed.2021.153579 33991865

[B93] Wang T. Guan R. Xia F. Du J. Xu L. (2021c). Curcumin promotes venous thrombi resolve process in a mouse deep venous thrombosis model *via* regulating miR-499. Microvasc. Res. 136, 104148. 10.1016/j.mvr.2021.104148 33631181

[B94] Wang Y. Zhou Y. Fu J. (2021d). Advances in antiobesity mechanisms of capsaicin. Curr. Opin. Pharmacol. 61, 1–5. 10.1016/j.coph.2021.08.012 34537583

[B95] Wang C. Yuan Z. Xie J. Lei Y. Li Y. Huang J. (2023). Integrated metabolomics and molecular docking reveal berberrubine inhibits thrombosis by regulating the vitamin K catalytic cycle in mice. Eur. J. Pharmacol. 938, 175436. 10.1016/j.ejphar.2022.175436 36481237

[B96] Wei X. Zhang B. Wei F. Ding M. Luo Z. Han X. (2022). Gegen Qinlian pills alleviate carrageenan-induced thrombosis in mice model by regulating the HMGB1/NF-κB/NLRP3 signaling. Phytomedicine 100, 154083. 10.1016/j.phymed.2022.154083 35413645 PMC9759718

[B97] Wen L. He T. Yu A. Sun S. Li X. Wei J. (2021). Breviscapine: a review on its phytochemistry, pharmacokinetics and therapeutic effects. Am. J. Chin. Med. 49 (6), 1369–1397. 10.1142/S0192415X21500646 34263720

[B98] Xie Z. Liu X. Huang X. Liu Q. Yang M. Huang D. (2021). Remodelling of gut microbiota by Berberine attenuates trimethylamine N-oxide-induced platelet hyperreaction and thrombus formation. Eur. J. Pharmacol. 911, 174526. 10.1016/j.ejphar.2021.174526 34599914

[B99] Xie J. Liao Y. Wang D. (2025). Baicalin promotes migration and angiogenesis of endothelial progenitor cells but impedes thrombus formation *via* SIRT1/NF-κB signaling in a rat model of deep vein thrombosis. Histol. Histopathol. 40 (4), 547–554. 10.14670/HH-18-799 39290181

[B100] Ye S. Liu X. Mao B. Yang L. Liu N. (2016). Paeonol enhances thrombus recanalization by inducing vascular endothelial growth factor 165 *via* ERK1/2 MAPK signaling pathway. Mol. Med. Rep. 13 (6), 4853–4858. 10.3892/mmr.2016.5135 27082415

[B101] Ye Y. Yang L. Leng M. Wang Q. Wu J. Wan W. (2023). Luteolin inhibits GPVI-mediated platelet activation, oxidative stress, and thrombosis. Front. Pharmacol. 14, 1255069. 10.3389/fphar.2023.1255069 38026984 PMC10644720

[B102] Yin Q. Zhang X. Liao S. Huang X. Wan C. C. Wang Y. (2023). Potential anticoagulant of traditional chinese medicine and novel targets for anticoagulant drugs. Phytomedicine 116, 154880. 10.1016/j.phymed.2023.154880 37267694

[B103] Yuan Y. Zhou X. Wang Y. Wang Y. Teng X. Wang S. (2020). Cardiovascular modulating effects of magnolol and Honokiol, two polyphenolic compounds from traditional Chinese medicine-magnolia Officinalis. Curr. Drug Targets 21 (6), 559–572. 10.2174/1389450120666191024175727 31749425

[B104] Zeng B. Wei A. Zhou Q. Yuan M. Lei K. Liu Y. (2022). Andrographolide: a review of its pharmacology, pharmacokinetics, toxicity and clinical trials and pharmaceutical researches. Phytother. Res. 36 (1), 336–364. 10.1002/ptr.7324 34818697

[B105] Zhang L. Wei W. (2020). Anti-inflammatory and immunoregulatory effects of paeoniflorin and total glucosides of paeony. Pharmacol. Ther. 207, 107452. 10.1016/j.pharmthera.2019.107452 31836457

[B106] Zhang Y. Du X. Li W. Sang H. Qian A. Sun L. (2018). Resveratrol improves endothelial progenitor cell function through miR-138 by targeting Focal Adhesion Kinase (FAK) and promotes thrombus resolution *in vivo* . Med. Sci. Monit. 24, 951–960. 10.12659/msm.906116 29447140 PMC5822936

[B107] Zhang J. Chen Z. Huang X. Shi W. Zhang R. Chen M. (2019a). Insights on the multifunctional activities of magnolol. Biomed. Res. Int. 2019, 1847130. 10.1155/2019/1847130 31240205 PMC6556366

[B108] Zhang L. Li D. C. Liu L. F. (2019b). Paeonol: pharmacological effects and mechanisms of action. Int. Immunopharmacol. 72, 413–421. 10.1016/j.intimp.2019.04.033 31030097

[B109] Zhang J. Wu C. Gao L. Du G. Qin X. (2020). Astragaloside IV derived from Astragalus membranaceus: a research review on the pharmacological effects. Adv. Pharmacol. 87, 89–112. 10.1016/bs.apha.2019.08.002 32089240

[B110] Zhang L. X. Li C. X. Kakar M. U. Khan M. S. Wu P. F. Amir R. M. (2021). Resveratrol (RV): a pharmacological review and call for further research. Biomed. Pharmacother. 143, 112164. 10.1016/j.biopha.2021.112164 34649335

[B111] Zhang X. X. Zuo J. Q. Wang Y. T. Duan H. Y. Yuan J. H. Hu Y. H. (2022a). Paeoniflorin in Paeoniaceae: distribution, influencing factors, and biosynthesis. Front. Plant Sci. 13, 980854. 10.3389/fpls.2022.980854 36119574 PMC9478390

[B112] Zhang Y. Ma C. He L. Liao L. Guo C. Wang C. (2022b). Tetramethylpyrazine protects endothelial injury and antithrombosis *via* antioxidant and antiapoptosis in HUVECs and zebrafish. Oxid. Med. Cell Longev. 2022, 2232365. 10.1155/2022/2232365 35898617 PMC9313999

[B113] Zhang H. J. Fu J. Yu H. Xu H. Hu J. C. Lu J. Y. (2024a). Berberine promotes the degradation of phenylacetic acid to prevent thrombosis by modulating gut microbiota. Phytomedicine 128, 155517. 10.1016/j.phymed.2024.155517 38518650

[B114] Zhang W. Zhang Y. Fan J. Feng Z. Song X. (2024b). Pharmacological activity of capsaicin: mechanisms and controversies (Review). Mol. Med. Rep. 29 (3), 38. 10.3892/mmr.2024.13162 38240083 PMC10828990

[B115] Zhang Y. Xin G. Zhou Q. Yu X. Feng L. Wen A. (2024c). Elucidating the distinctive regulatory effects and mechanisms of active compounds in Salvia miltiorrhiza Bunge *via* network pharmacology: unveiling their roles in the modulation of platelet activation and thrombus formation. Toxicol. Appl. Pharmacol. 484, 116871. 10.1016/j.taap.2024.116871 38423217

[B116] Zhang S. Liu C. Li W. Zhang Y. Yang Y. Yang H. (2025). Kaempferol promotes angiogenesis through HIF-1α/VEGF-A/Notch1 pathway in ischemic stroke rats. Neurochem. Int. 185, 105953. 10.1016/j.neuint.2025.105953 39988285

[B117] Zhao Z. Nian M. Qiao H. Yang X. Wu S. Zheng X. (2022). Review of bioactivity and structure-activity relationship on baicalein (5,6,7-trihydroxyflavone) and wogonin (5,7-dihydroxy-8-methoxyflavone) derivatives: structural modifications inspired from flavonoids in Scutellaria baicalensis. Eur. J. Med. Chem. 243, 114733. 10.1016/j.ejmech.2022.114733 36155355

[B118] Zhu M. Sun Y. Su Y. Guan W. Wang Y. Han J. (2024). Luteolin: a promising multifunctional natural flavonoid for human diseases. Phytother. Res. 38 (7), 3417–3443. 10.1002/ptr.8217 38666435

